# Sterol metabolism regulates neuroserpin polymer degradation in the absence of the unfolded protein response in the dementia FENIB

**DOI:** 10.1093/hmg/ddt310

**Published:** 2013-06-28

**Authors:** Benoit D. Roussel, Timothy M. Newton, Elke Malzer, Nikol Simecek, Imran Haq, Sally E. Thomas, Marian L. Burr, Paul J. Lehner, Damian C. Crowther, Stefan J. Marciniak, David A. Lomas

**Affiliations:** Department of Medicine, University of Cambridge, Cambridge Institute for Medical Research (CIMR), Wellcome Trust/MRC Building, Hills Road, Cambridge CB2 0XY, UK

## Abstract

Mutants of neuroserpin are retained as polymers within the endoplasmic reticulum (ER) of neurones to cause the autosomal dominant dementia familial encephalopathy with neuroserpin inclusion bodies or FENIB. The cellular consequences are unusual in that the ordered polymers activate the ER overload response (EOR) in the absence of the canonical unfolded protein response. We use both cell lines and *Drosophila* models to show that the G392E mutant of neuroserpin that forms polymers is degraded by UBE2j1 E2 ligase and Hrd1 E3 ligase while truncated neuroserpin, a protein that lacks 132 amino acids, is degraded by UBE2g2 (E2) and gp78 (E3) ligases. The degradation of G392E neuroserpin results from SREBP-dependent activation of the cholesterol biosynthetic pathway in cells that express polymers of neuroserpin (G392E). Inhibition of HMGCoA reductase, the limiting enzyme of the cholesterol biosynthetic pathway, reduced the ubiquitination of G392E neuroserpin in our cell lines and increased the retention of neuroserpin polymers in both HeLa cells and primary neurones. Our data reveal a reciprocal relationship between cholesterol biosynthesis and the clearance of mutant neuroserpin. This represents the first description of a link between sterol metabolism and modulation of the proteotoxicity mediated by the EOR.

## INTRODUCTION

Neuroserpin is a member of the SERPIN super family of serine protease inhibitors (1). It is mainly synthesized and secreted from neurones of the central and peripheral nervous systems (2,3), but it has also been detected in the heart, kidney, spinal cord and testis (3). Neuroserpin is believed to inhibit tissue-type plasminogen activator (tPA) (4) and plays an important role in physiological processes such as synaptic plasticity (2,5) and memory (6,7) and in the pathogenesis of neurological disorders mediated by excitotoxicity, such as stroke (2,8–11). Through interaction with the low-density lipoprotein receptor-related protein, neuroserpin may also mediate internalization of tPA complexes to regulate its proteolytic activity (12). Although mice engineered to lack expression of neuroserpin do not display altered tPA activity in the brain, they do exhibit altered emotional responses (13).

Mutations in neuroserpin underlie the autosomal dominant dementia familial encephalopathy with neuroserpin inclusion bodies (FENIB) (14–16). This dementia is characterized by eosinophilic neuronal inclusions of neuroserpin (Collins bodies) within the deeper layers of the cerebral cortex and *substantia nigra*. Six mutations of neuroserpin have been identified in FENIB resulting in a spectrum of symptoms from dementia in middle age to severe epilepsy under the age of 10 years (17). The mutations (Ser49Pro, Leu47Pro, Ser52Arg, His338Arg, Gly392Arg and Gly392Glu) cluster in the shutter domain of neuroserpin and result in the formation of ordered polymers within the endoplasmic reticulum (ER) (18). They demonstrate a clear genotype–phenotype correlation, with mutations that cause more rapid polymerization of the protein causing earlier onset disease (18). These inclusions cause disease both by gain-of-function toxicity and loss of function of an important protease inhibitor (18). One of the striking features of FENIB is that the ER inclusions of mutant neuroserpin do not activate the unfolded protein response (UPR) (8,19,20). Instead, they appear to trigger NF-κB signalling through release of calcium from the ER, which has been termed the ‘ER overload response’ (EOR) (19). However, before it can form polymers, the majority of mutant neuroserpin appears to be degraded predominantly by the ER-associated degradation (ERAD) pathway, although autophagy may also contribute to this (20). Many ERAD components are regulated as part of the UPR; however, how ERAD can be regulated in a disorder that does not cause ER stress is unknown.

We report here a critical role for HMGCoA reductase, the limiting enzyme of the cholesterol biosynthetic pathway, in the clearance of mutant neuroserpin from the ER. This pathway is upregulated in cells expressing polymers of neuroserpin. While the interrelationship between the UPR and sterol metabolism is well recognized, this represents the first description of a link between sterol metabolism and modulation of the proteotoxicity mediated by the EOR.

## RESULTS

### Generation of a human cell model of FENIB

**Figure 1.**
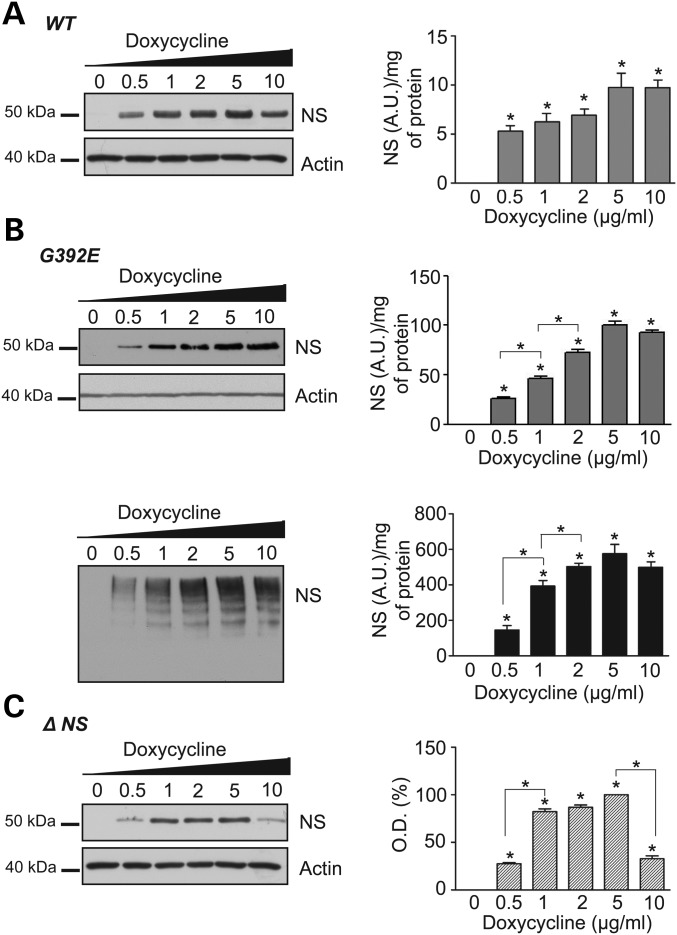
Characterization of HeLa cells that conditionally express neuroserpin. (**A**) 10% w/v acrylamide SDS–PAGE and western blot analysis (left) and ELISA (right) for total neuroserpin to assess the concentration response to doxycycline of cells that conditionally express wild-type neuroserpin. (**B**) 10% w/v acrylamide SDS–PAGE and western blot analysis (top left) and ELISA (top right) for total neuroserpin to assess the concentration response to doxycycline of cells that conditionally express G392E neuroserpin. 7.5% w/v acrylamide non-denaturing-PAGE and western blot analysis (bottom left) for total neuroserpin to assess the formation of polymers. ELISA (bottom right, black histogram) for polymers of G392E neuroserpin detected with the 7C6 monoclonal antibody. (**C**) 10% w/v acrylamide SDS–PAGE and western blot analysis (left) and associated densities (right) for total neuroserpin to assess the concentration response to doxycycline of cells that conditionally express Δ neuroserpin. The data in (A)–(C) are from three independent experiments, *P < 0.05.

**Figure 2.**
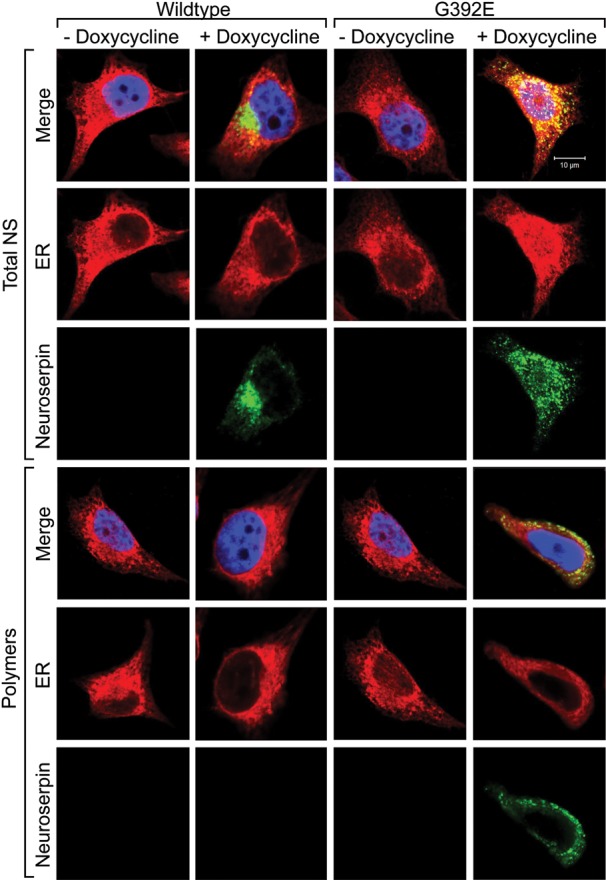
The G392E mutant of neuroserpin is retained in the ER. Confocal microscopy of HeLa cells cultured for 48 h in the presence or absence of 2 µg/ml doxycycline. Wild-type or G392E neuroserpin cells are stained for total or polymers of neuroserpin with the 1A10 or 7C6 antibody, respectively (green). The ER-resident protein calreticulin is stained red. Merged images (yellow) correspond to areas with overlapping red and green staining, showing co-localization of neuroserpin and the ER. The nucleus is blue with Hoerscht stain. All images are shown at the same scale, with the exception of wild-type neuroserpin cells in the presence of doxycycline, stained for total neuroserpin.

A human cell model of FENIB was developed by expressing neuroserpin under the control of the Tet-On promotor in HeLa cells. Clonal cell lines were generated that conditionally express either wild-type neuroserpin (Fig. 1A), the naturally occurring G392E mutant that forms ordered polymers in association with FENIB (G392E cells) (Fig. 1B) or a synthetic truncation, Δ neuroserpin, lacking the C-terminal 132 amino acids (Δ cells) that we have previously used as a positive control for ER stress (19,20) (Fig. 1C). In each cell line, doxycycline induced the expression of neuroserpin in a concentration-dependent manner as assessed by western blot analyses and ELISA (Fig. 1). Treatment with doxycycline at 2 µg/ml for 48 h was considered optimal, providing a useful dynamic range of neuroserpin expression. Δ neuroserpin could be detected only by western blot analysis as the monoclonal antibodies used in the ELISA give little signal for the truncated protein. The ELISA detected either ‘total neuroserpin’ with a pool of monoclonal antibodies [1A10, 10B8, 10G12 (18)] or polymers of G392E neuroserpin with the 7C6 antibody (18). Cells expressing wild-type neuroserpin stained positively for neuroserpin within the ER and Golgi apparatus, as is typical of a secretory protein, whereas G392E neuroserpin formed inclusion bodies of polymers within the ER that recapitulate the features of FENIB (Fig. 2).

The next step in the characterization of our human cell-inducible model of FENIB was to understand the mechanism of degradation of neuroserpin mutants.

### Identification of E3 and E2 ligases involved in the degradation of mutants of neuroserpin

**Figure 3.**
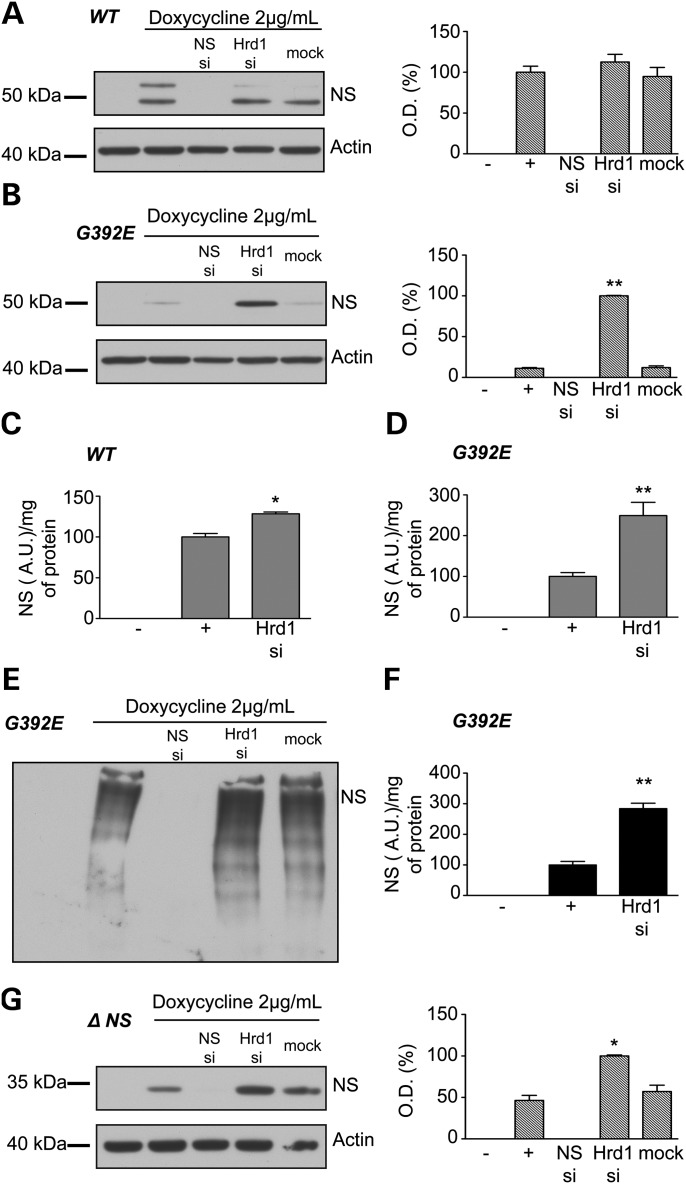
Effect of the knockdown of ubiquitin-E3 ligase Hrd1 on neuroserpin levels in HeLa cells. HeLa inducible cell lines were treated with 2 µg/ml doxycycline and transfected or not with neuroserpin siRNA as a control of transfection, Hrd1 siRNA or a mock transfection for 2 days. The silencing of Hrd1 in wild-type cells does not affect the level of neuroserpin as seen by western blot analysis and densitometry (hatched histogram) (**A**) and has only a small effect when assessed by ELISA against total neuroserpin (**C**). Mock transfection does not affect the level of neuroserpin, while siRNA targeting neuroserpin abolishes its expression. The level of neuroserpin increases when Hrd1 is silenced in cells expressing G392E neuroserpin, as shown by western blot analysis and associated densitometries (hatched histogram) (**B**) or by ELISA against total neuroserpin (**D**). Western blot analysis of non-denaturing PAGE (**E**) and ELISA against polymers of neuroserpin (**F**) demonstrate that the increase in G392E neuroserpin is associated with an increase in polymers. The truncated protein Δ neuroserpin also accumulates in cells when Hrd1 is silenced, but at a lower level when compared with G392E neuroserpin (**G**). *n* = 4 independent repeats for each cell line. *P < 0.05; **P < 0.005.

The E3 ligases Hrd1 and gp78 have been implicated in the degradation of various serpins including Alpha-1-antitrypsin (21) and neuroserpin (22). We decided to investigate the specific role of these ligases in the degradation of neuroserpin. By using a *Drosophila melanogaster* model of FENIB, we confirmed the crucial role of these two E3 ligases in ERAD (Supplementary Material, Fig. S1). Then, we tested the depletion of Hrd1 and gp78 in our cells. Silencing of *Hrd1* expression was achieved by transfection with siRNA. While this did not affect the level of wild-type neuroserpin as detected by western blot analysis (Fig. 3A), there was a small but significant increase detected by ELISA (Fig. 3C). In contrast, a control siRNA to *neuroserpin* abolished its expression in all cell lines (Fig. 3A, B, E, G). The double band of immunoreactive neuroserpin observed intermittently in cells expressing wild-type protein (Fig. 3A) is probably a degradation product as wild-type neuroserpin can be cleaved by proteases such as tPA. In cells expressing G392E neuroserpin, depletion of *Hrd1* resulted in a striking increase in the level of mutant neuroserpin within the cell, as seen by western blot analysis (Fig. 3B) and ELISA against total neuroserpin (Fig. 3D). This reflected an increase in polymerized neuroserpin as shown both by non-denaturing PAGE (Fig. 3E) and polymer-specific ELISA (Fig. 3F). A similar, albeit less pronounced, increase in neuroserpin was also observed in cells expressing Δ neuroserpin (Fig. 3G).

**Figure 4.**
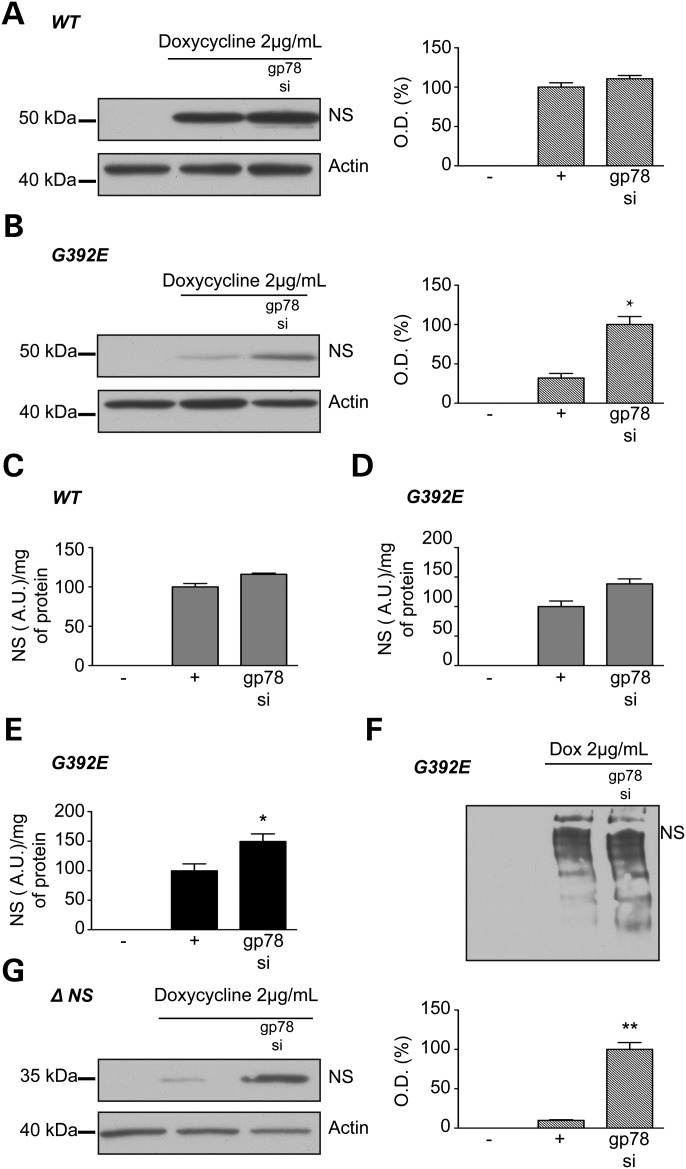
Effect of the knockdown of the ubiquitin-E3 ligase gp78 on neuroserpin in HeLa cells. HeLa inducible cell lines were treated with 2 µg/ml doxycycline and transfected or not with gp78 siRNA for 2 days. There was no change in the amount of wild-type neuroserpin when assessed by western blot analysis and associated densitometry (hatched histogram) (**A**) or by ELISA against total neuroserpin (**C**). A small increase in G392E neuroserpin was seen when gp78 was silenced as assessed by western blot analysis and associated densitometry (hatched histogram) (**B**), but this increase was not statically different when assessed by ELISA against total neuroserpin (**D**). However, there was a statistically significant increase in G392E neuroserpin polymers when measured by ELISA (**E**). Western blot analysis of non-denaturing PAGE also shows a small increase in polymers when gp78 is silenced (**F**). Silencing of gp78 in cells expressing Δ neuroserpin resulted in a dramatic increase in protein accumulation as shown by western blot analysis and associated densitometry (**G**). *n* = 4 independent repeats for each cell line. *P < 0.05 ; **P < 0.005.

Knock down of *gp78* had no effect on the expression of wild-type neuroserpin when assessed either by western blot analysis (Fig. 4A) or ELISA for total neuroserpin (Fig. 4C). In contrast, depletion of *gp78* increased the level of polymers in cells expressing G392E neuroserpin when assessed by ELISA without affecting the total level of neuroserpin (Fig. 4B and D–F). Finally, knock down of *gp78* markedly increased the level of truncated Δ neuroserpin (Fig. 4G).

Hrd1 and gp78 knock down were processed on the same ELISA plates, allowing a direct comparison. From our data (western blot analysis and ELISA), we concluded that Hrd1 plays an important role in the degradation of G392E polymeric neuroserpin, while gp78 is preferentially involved in the degradation of misfolded Δ neuroserpin despite a role for both of them in the degradation of polymerogenic and truncated neuroserpin.

**Figure 5.**
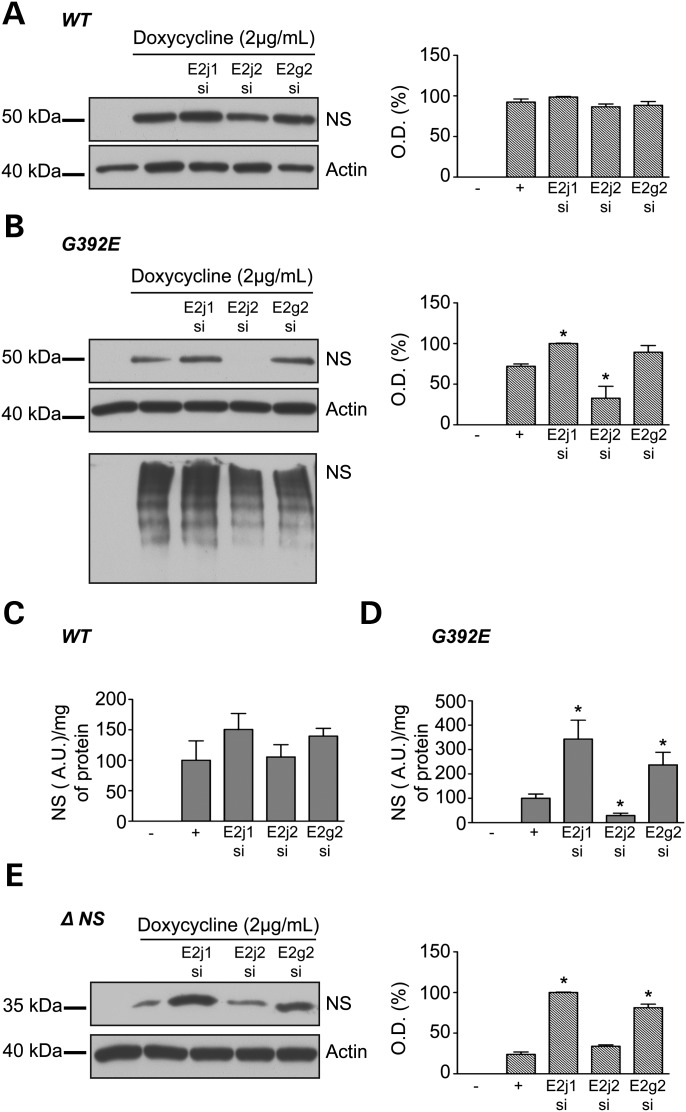
Effect of the knockdown of ubiquitin-E2 ligases on neuroserpin levels in HeLa cells. HeLa cells were induced with 2 µg/ml doxycycline and transfected with ubiquitin-E2 ligase (UBE2) siRNA for 2 days. Depletion of UBE2j1, UBE2j2 or UBE2g2 did not affect the level of wild-type neuroserpin as assessed by western blot analysis and associated densitometry (hatched histogram) (**A**) and by ELISA against total neuroserpin (**C**). In contrast, the depletion of UBE2j1 results in an increase in neuroserpin polymers in cells expressing G392E neuroserpin as observed by western blot analysis and densitometry (**B**) and by ELISA (**D**). Silencing of UBE2g2 resulted in a slight increase in neuroserpin levels that was only statically different in ELISA (D). UBE2j2 silencing leads to a decrease in neuroserpin polymer level in HeLa cells as seen by western blot analysis (B) and ELISA (D). In cells expressing Δ neuroserpin silencing both UBE2j1 and UBE2j2 result in an increase in neuroserpin levels, whereas the depletion of UBE2j2 has no effect (**E**). *n* = 4 independent repeats for each cell line. *P < 0.05.

The *Drosophila melanogaster* model also suggested the involvement of the E2 ligase UBE2j1 (Supplementary Material, Fig. S1). To test this in a mammalian system, we depleted our cells of *UBE2j1*, the E2 ubiquitin ligase of Hrd1, and *UBE2g2*, the E2 ligase of gp78 (23–25). *UBE2j2*, an E2 ligase that interacts with the E3 ligase mK3 (26), served as a control. Depletion of *UBE2j1*, *UBE2j2* or *UBE2g2* did not affect the level of wild-type neuroserpin as assessed by western blot analysis or ELISA for total neuroserpin (Fig. 5A and C). In contrast, the depletion of *UBE2j1* resulted in an increase in neuroserpin polymers in cells expressing G392E neuroserpin as observed by western blot analysis and by ELISA (Fig. 5B and D). Silencing of *UBE2g2* resulted in an increase in neuroserpin levels (Fig. 5D). Surprisingly, silencing of *UBE2j2* decreased the levels of neuroserpin polymers as determined either by western blot analysis or ELISA (Fig. 5B and D). Silencing of either *UBE2j1* or *UBE2j2* increased levels of Δ neuroserpin, whereas the depletion of *UBE2j2* had no effect (Fig. 5E). None of the siRNA treatments affected cell viability or the level of ER chaperones (data not shown). To summarize, we observed the UBE2J1 ligase couple to be involved in the degradation of well-ordered polymers of neuroserpin, while the UBE2g2/gp78 ligase complex appears to be responsible for the degradation of misfolded Δ neuroserpin.

Since we demonstrated that the UPR is not activated in FENIB (19), we investigated the activation of alternative pathways that could lead to the activation of ERAD.

### Transcriptional analysis of cells expressing polymers of neuroserpin reveals upregulation of the cholesterol biosynthetic pathway

**Figure 6.**
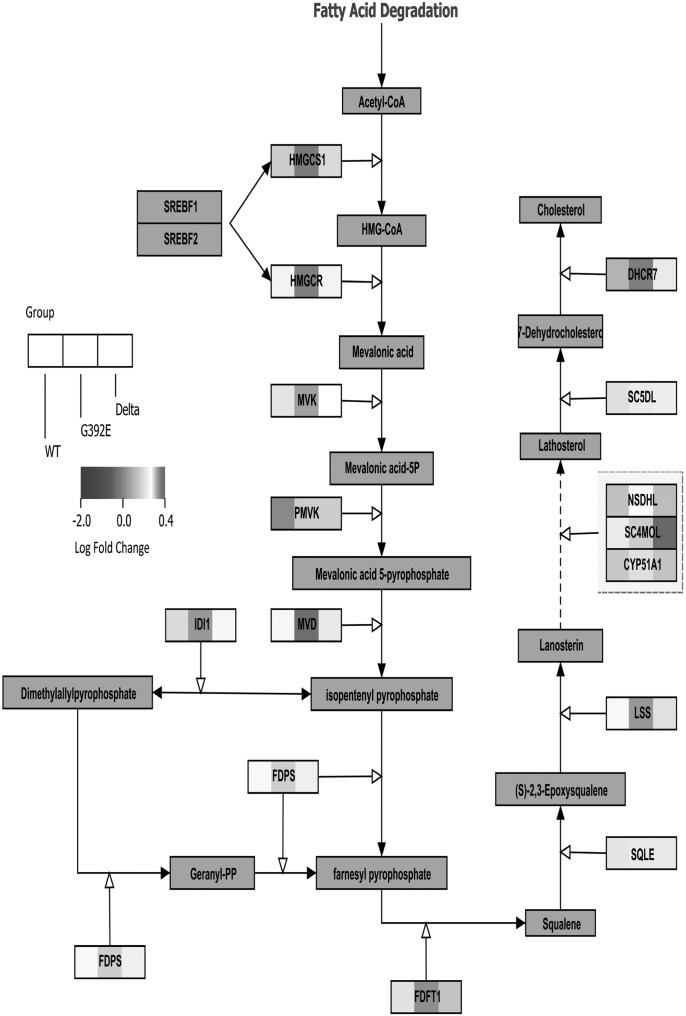
Whole genome and gene set enrichment analysis of cells expressing mutants of neuroserpin show an upregulation of the cholesterol pathway when polymers are expressed. Fold changes representation in the cholesterol biosynthesis pathway of cells expressing wild-type, G392E or Δ neuroserpin based on the microarray analysis (GEO number GSE46230) followed by a Gene Set Enrichment analysis (27).

In order to identify signals initiated by the polymerization of neuroserpin within the ER, we next used oligonucleotide microarrays to perform a transcriptional analysis of cells expressing wild-type, G392E or Δ neuroserpin. Treatment of Tet-On cells with 2 µg/ml of doxycycline increased the level of wild-type neuroserpin mRNA by 3.75 log-fold, G392E neuroserpin by 3.53 log-fold and Δ neuroserpin by 5.24-log fold (GEO accession number: GSE46230, and full analysis on Supplementary Material, Fig. S2). Gene Set Enrichment analysis (27) was performed on the normalized gene expression values for each cell line (see Materials and Methods). Fifty-four pathways were upregulated when wild-type neuroserpin was expressed (Supplementary Material, Table S1), while 70 pathways were upregulated following the expression of G392E neuroserpin (Supplementary Material, Table S2). No pathways were upregulated with a FDR ≤ 0.25 following expression of Delta neuroserpin. Unique pathway analysis of the G392E neuroserpin cell line revealed the cholesterol biosynthetic pathway to be modulated. This has previously been linked with the responses to ER dysfunction and to ERAD (28–30). Since most genes of this pathway were upregulated in cells expressing G392E neuroserpin but not in cells expressing the wild-type or Δ neuroserpin, we chose to examine this response further (Fig. 6).

To validate the Gene Set Enrichment analysis, RT-qPCR was performed to assess the expression of four key genes in the cholesterol biosynthetic pathway (HMGCoA reductase, HMGCoA synthase, mevalonate (diphospho) decarboxylase and lanosterol synthase) in cells prior to and following induction of neuroserpin expression. HMGCoA reductase, HMGCoA synthase and mevalonate (diphospho) decarboxylase were upregulated in cells expressing polymers of G392E neuroserpin (Supplementary Material, Fig. S3). Only HMGCoA reductase was increased following the expression of Δ neuroserpin. In order to test the physiological relevance of this finding, we next set out to measure the activation of the cholesterol biosynthetic pathway.

### Polymers of neuroserpin activate the cholesterol biosynthetic pathway

**Figure 7.**
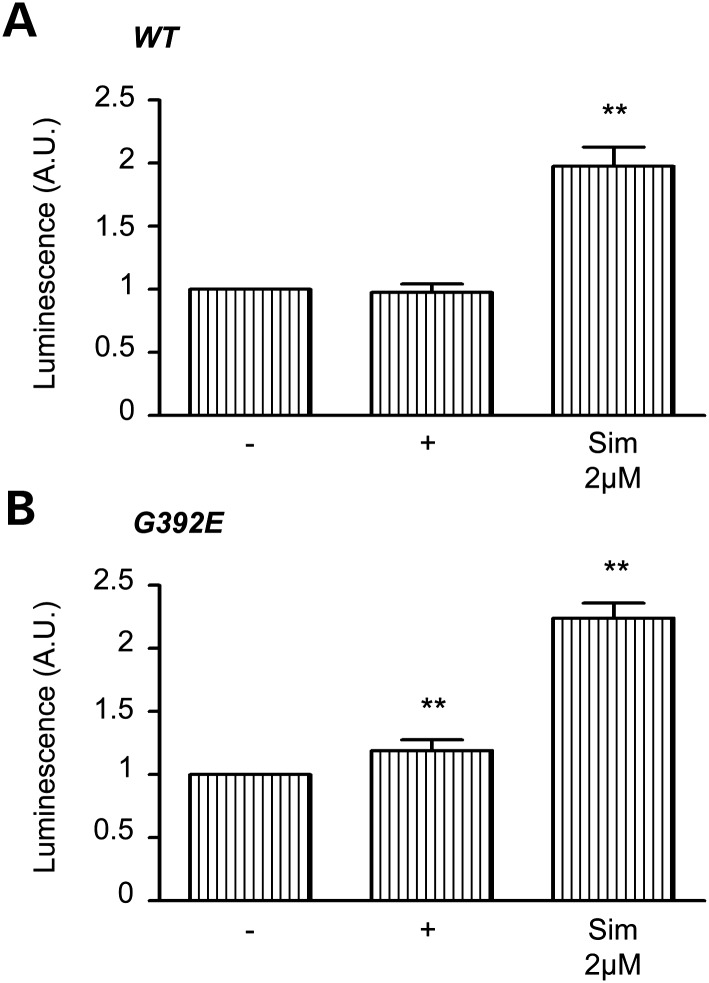
Polymers of neuroserpin activate the cholesterol biosynthesis pathway in HeLa cells. HeLa cells expressing wild-type (**A**) or G392E (**B**) neuroserpin were induced with 2 μg/ml doxycycline or 5 µm simvastatin for 48 h before collection. SREBP2 activation is indicated by the Luciferase activity of a SREBP reporter plasmid measured relative to a Renilla luciferase control plasmid. Histograms represent the mean of *n* = 5. **P < 0.005.

The transcriptional upregulation of genes in the cholesterol biosynthetic pathway is mediated by the SREBP transcription factor (31). To investigate how the upregulation of this pathway occurred during the expression of neuroserpin, we generated a reporter of SREBP2 signalling by fusing the promoter region of *Cricetulus griseus* HMG synthase to the pGL2 luciferase reporter plasmid (Fig. 7A and B). In the presence of doxycycline, expression of wild-type neuroserpin did not result in SREBP2 activation (Fig. 7A), while G392E polymers of neuroserpin activated SREBP2 (Fig. 7B). Simvastatin, a potent inhibitor of HMGCoA reductase, was used as a positive control for the activation of SREBP2. Our data indicate that the expression of a polymerogenic mutant of neuroserpin, which are known to activate the EOR but not the UPR (19), also initiates SREBP2-dependent signalling.

### Inhibition of sterol synthesis leads to a decrease in neuroserpin ubiquitination, and to the accumulation of mutants of neuroserpin in both HeLa cells and primary neurones

**Figure 8.**
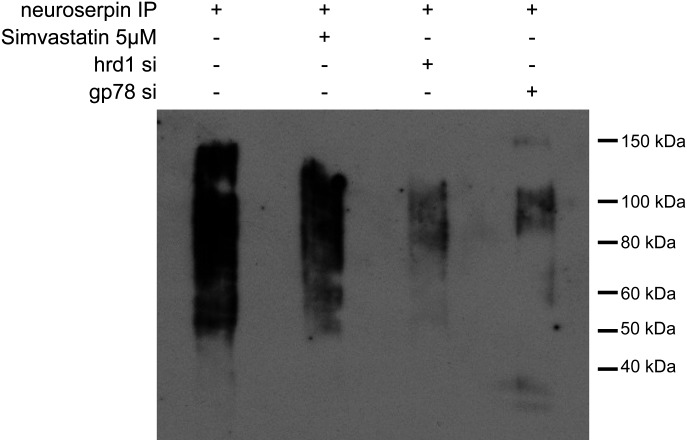
Degradation of polymers of neuroserpin is dependant of HMGCoA reductase. HeLa cells expressing G392E neuroserpin were induced with 2 µg/ml doxycycline for 2 days and treated with 40 µm of MG132 for the last 4 h of the experiment. These cells were treated with or without 5 µm simvastatin or transfected with Hrd1 or gp78 siRNA. Three milligrams of total protein was immunoprecipitated using a rabbit polyclonal antibody against neuroserpin, and then assessed by western blot analysis on a 10% w/v acrylamide SDS gel. The membrane was developed using the ubiquitin-P4D1 antibody. Ubiquitination of G392E neuroserpin was decreased by treatment with simvastatin. A reduction in ubiquitination was also observed when Hrd1 or gp78 are depleted by siRNA.

Since we had found that polymers of neuroserpin are able to activate the cholesterol biosynthetic pathway, we wished to determine if the degradation of these polymers was also dependent on this pathway. Treatment with simvastatin (32) significantly reduced the ubiquitination of the polymerogenic G392E neuroserpin. Similar results were found when either gp78 or Hrd1 were depleted by siRNA (Fig. 8).

**Figure 9.**
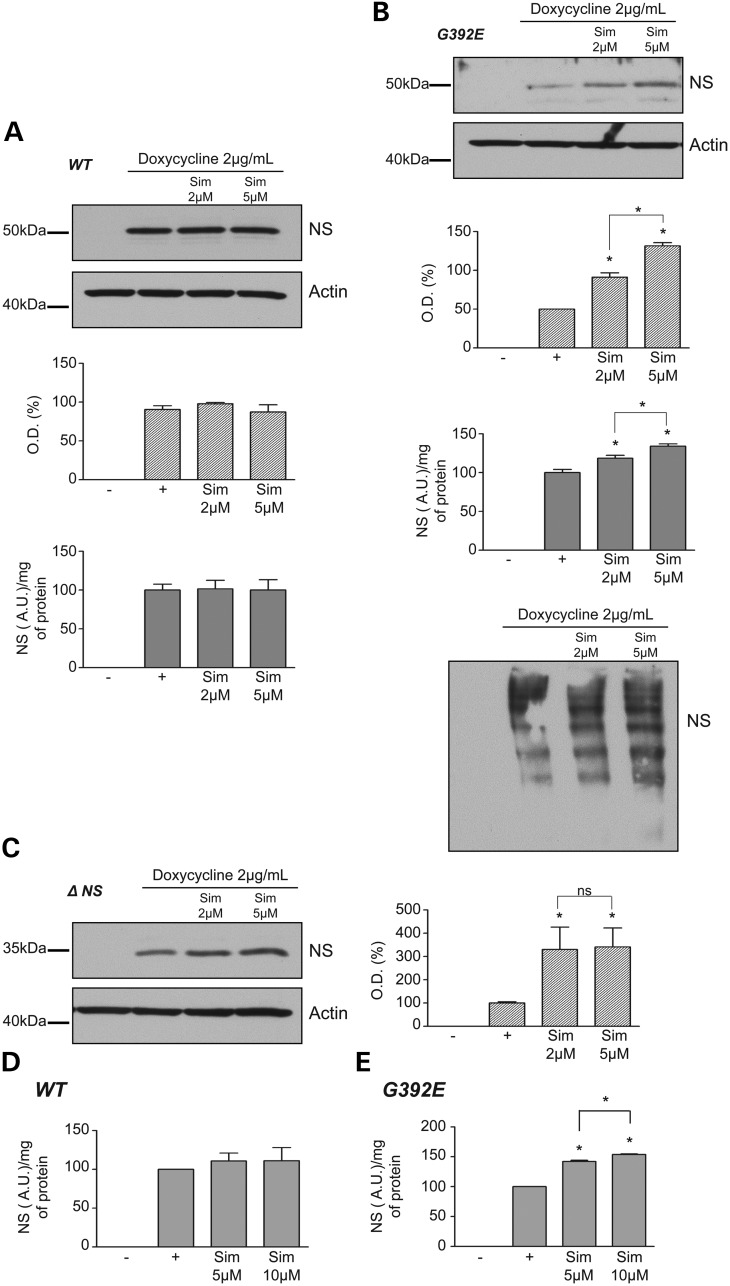
Inhibition of the cholesterol biosynthesis pathway leads to an accumulation of mutants of neuroserpin in both HeLa cells and primary neurones. Neuroserpin expression was induced in HeLa cells (**A**–**C**) with 2 μg/ml doxycycline and treated or not with simvastatin (2 or 5 µm) for 2 days. Cell lysates were resolved by 10% w/v acrylamide SDS- and 7.5% w/v acrylamide non-denaturing PAGE (for G392E neuroserpin) with associated-densitometries (hatched histograms) and measured by ELISA directed against total neuroserpin (grey histograms). The same membranes were stripped and re-probed to detect actin as a loading control. (A) The expression of wild-type neuroserpin was unaffected when cells were treated with 2 or 5 µm simvastatin for 2 days. Neuroserpin levels were detected by western blot analysis and densitometry and ELISA. (B) Treatment of HeLa cells expressing G392E neuroserpin with simvastatin results in a concentration-dependent increase in neuroserpin. This was apparent on western blot analysis of both SDS (and associated densitometries in hatched histogram) and by ELISA (grey histogram) for total neuroserpin. Non-denaturing PAGE confirmed that the protein was present as polymers. (C) Treatment with simvastatin increases the amount of Δ neuroserpin in HeLa cells when assessed by western blot analysis and associated densitometry, without a concentration response. Primary neurones were transfected with the wild-type (**D**) or G392E (**E**) neuroserpin after 7 days *in vitro* and treated or not with simvastatin. Forty-eight hours later, the neurones were lysed and assessed for total neuroserpin by ELISA. Simvastatin has no effect on wild-type neuroserpin (D) but increases the quantity of G392E neuroserpin in a concentration-dependent manner (E). Data are from three independent experiments for each cell line. *P < 0.05.

To understand the homeostatic function of enhanced sterol synthesis, we determined the consequences of inhibiting sterol metabolism in HeLa cells that express wild-type and mutant neuroserpin. No effect on neuroserpin levels was detected by western blot or ELISA when wild-type neuroserpin-expressing HeLa cells were treated with 2 and 5 µm simvastatin for 48 h (Fig. 9A). However, treatment with simvastatin resulted in a concentration-dependent accumulation of polymers in cells expressing G392E neuroserpin, as detected by western blot analysis (SDS and non-denaturing PAGE) and ELISA (Fig. 9B). Treatment with simvastatin also increased the level of Δ neuroserpin protein (Fig. 9C). In contrast, inhibition of squalene epoxidase by terbinofine, oxidosqualene-lanosterol cyclase by fumarate (Ro48–8071) and cytochrome P450 3A4 by ketoconazole did not alter the level of wild-type, G392E or Δ neuroserpin within cells (data not shown).

To determine if the observations made in cell lines held true for primary tissue, we next examined the relationship between sterol synthesis and the accumulation of neuroserpin polymers in primary cultures of mouse cortical neurones. Neurones were transfected either with wild-type neuroserpin or the G392E mutant and left untreated or treated with simvastatin for 48 h. The quantity of intracellular neuroserpin was then assessed by ELISA. Simvastatin had no effect on the quantity of wild-type neuroserpin (Fig. 9D), but increased the levels of G392E neuroserpin in a concentration-dependent manner (Fig. 9E).

## DISCUSSION

Mutants of neuroserpin are retained within the ER of neurones to cause the inclusion body dementia FENIB. This dementia is unusual as the ordered structure of neuroserpin polymers prevents an UPR (19). Instead, the accumulation of neuroserpin leads to an activation of the EOR (20). This provides a model to evaluate the cellular consequences of the retention of ordered protein within cells.

After creating a cellular model to investigate FENIB, we identified the E3 and E2 ligases involved in neuroserpin degradation. This was performed with the use of a Drosophila model of FENIB that shown accumulation of G392E neuroserpin when E3 and E2 orthologues were knocked down. It is interesting to note that the CG5623 depletion (UBE2j1 and j2) did not show any accumulation while in HeLa cells UBE2j1 shows a clear effect. These discrepancies between insects and mammals are well characterized in the literature, for example p53 is only implicated in cell death in flies while in mammals it has a role in the control of both cell death and cell cycle regulation (33). While a previous study has found the E3 ligases gp78 and hrd1 to be involved in neuroserpin degradation (22), we identified differences in the specificity of these proteins. Gp78, and its partner E2 ligase UBE2g2, target unfolded, truncated forms of neuroserpin, while hrd1 and UBE2j1 target ordered polymers of neuroserpin for degradation by ERAD. Hrd1 (21,34) and gp78 (35) have also been well-characterized in the degradation process of mutant alpha-1-antitrypsin and many other misfolded proteins (36,37).

Microarray analysis, and subsequent quantitative PCR, revealed that the expression of ordered polymers was associated with an activation of the sterol biosynthesis pathway. Previous studies have shown an interaction between ERAD and cholesterol biosynthetic pathway (38–40). HMGCoA reductase, the rate limiting enzyme in cholesterol biosynthesis, can interact with the E3 ligase gp78 (28) to mediate its sterol-accelerated degradation. Moreover, *in vitro* work has demonstrated that Hrd1 is involved in the dislocation and the degradation of HMGCoA reductase (41). From our experiments, we believe that accumulation of mutant neuroserpin leads to the activation of the cholesterol biosynthetic pathway. However, only inhibition of HMGCoA reductase and not other enzymes on this pathway increases the retention of mutant neuroserpin; HMGCoA reductase is the rate limiting enzyme of the pathway and so it is likely that accumulation of mutant neuroserpin increases HMGCoA reductase, and hence upregulates the whole pathway.

An interaction between the cholesterol biosynthetic pathway and ERAD has previously been observed in another neurological disease. In patients with Gaucher's disease, the level of intracellular cholesterol appears to modify the degradation of glucocerebrosidase by influencing ERAD (29); a reduced level of cholesterol biosynthesis leading to less glucocerebrosidase entering the ERAD pathway. Similarly, in the case of neuroserpin, we found that inhibition of cholesterol biosynthesis also reduced degradation of the pathogenic target protein. Importantly, however, whereas the activation of ERAD by misfolding glucocerebrosidase may involve ER stress (19), this is absent during the accumulation of neuroserpin polymers indicating that a sterol-independent mechanism can mediate this response.

Taken together, these experiments reveal a reciprocal relationship between cholesterol biosynthesis and the clearance of mutant neuroserpin. The accumulation of intracellular polymers of neuroserpin leads to the induction of genes involved in cholesterol synthesis, while inhibition of sterol biosynthesis impairs the ubiquitination of mutant neuroserpin and thus causes its accumulation.

## MATERIALS AND METHODS

### Reagents

Unless stated otherwise, reagents, buffers, culture media and serum for cell cultures were from Sigma-Aldrich Co (Dorset, UK). Custom-made rabbit polyclonal anti-neuroserpin antibody was produced in our laboratory (42,43). Antibodies were purchased as follow: mouse monoclonal anti-actin antibody (Abcam), α-Ub P4D1 (Santa Cruz Biotechnology), rabbit α-HRD1 (Abgent), mouse α-KDEL (Enzo life science), goat polyclonal anti-rabbit IgG (HRP) and rabbit polyclonal anti-mouse IgG (HRP) antibodies (Sigma-Aldrich Co). siRNA gene depletions were performed using a pool of four ONTARGETplus oligonucleotides (Dharmacon) and the GADD34siRNA as a scramble control. Simvastatin and MG132 were from Calbiochem; terbinofine, fumarate and ketoconazole from Sigma.

### Construction of neuroserpin expression plasmids

Plasmids expressing human wild-type, G392E and Δ neuroserpin (truncated protein) are described in Ref. (42). These constructs were subcloned into the pTRE-Tight vector (Clontech, Saint-Germain-en-Laye, France) for the generation of the stable HeLa Tet-On neuroserpin cell lines.

### Generation and characterization of stable Tet-On HeLa cells lines expressing neuroserpin

The HeLa Tet-On cell line was purchased from Clontech. Stable HeLa-neuroserpin cell lines were generated as detailed by the manufacturer and screened for neuroserpin expression by western blot analysis, ELISA and immunostaining. The cells were cultured in DMEM supplemented with 10% v/v Tet-free approved FBS (Clontech), 200 µg/ml Geneticin and 100 µg/ml Hygromycin B (both selective antibiotics from Invitrogen, Paisley, UK), at 37°C and 5% v/v CO_2_ in a humidified incubator. Neuroserpin expression was typically induced for 2 days by treatment with doxocycline (Clontech). SDS and non-denaturing PAGE followed by western-blot analysis and immunocytochemistry for confocal microscopy were performed as described previously (43).

### Primary culture of neurones and transfection

Neuronal cultures were prepared from Swiss mouse embryos (embryonic days 15–16) as described in Ref. (44). Cortices were dissected and dissociated in DMEM, and plated on 24-well plates previously coated with poly-d-lysine (0.1 mg/ml) (Sigma) and laminin (0.02 mg/ml) (Fisher Scientific). Cells were cultured in DMEM supplemented with 5% v/v fetal bovine serum, 5% v/v horse serum (both from Invitrogen) and 2 mm glutamine. Cultures were maintained at 37°C in a humidified 5% v/v CO_2_ atmosphere. Cytosine β-d-arabinoside (10 μm) was added after 3 days *in vitro* (DIV) to inhibit glial proliferation. At 7DIV, cells were transfected with plasmids expressing wild-type or G392E neuroserpin (42) using the Lipofectamine 2000 protocol (Invitrogen) with a transfection efficiency of ∼20%, as assessed by a GFP transfection control (data not shown). Treatment with simvastatin was performed the same day and cells were harvested for assessment by ELISA 2 days after transfection.

### Sandwich ELISA

Unless stated otherwise, all steps were carried out at room temperature in 50 µl per well. Plates (Corning Inc., Costar 3590) were coated overnight at 4°C with antigen-purified rabbit polyclonal anti-neuroserpin antibody at 2 µg/ml in PBS. Next, wells were washed with 0.9% w/v NaCl, 0.05% v/v Tween20 and blocked for 2 h with 300 µl/well of blocking buffer (PBS, 0.25% w/v BSA, 0.05% v/v Tween20, 0.025% w/v Na azide). Standards (recombinant purified wild-type or polymerized mutant neuroserpin) and unknown samples (cell lysates prepared as for western-blot analysis or fly extracts) were diluted in blocking buffer and incubated for 2 h. After washing, the wells were incubated with either a pool of monoclonal antibodies (1A10, 10B8 and 10G12, each 333 ng/ml) to detect total neuroserpin or with an individual monoclonal antibody (7C6 1 µg/ml) to detect polymers of neuroserpin, diluted in blocking buffer for 2 h. Bound monoclonal antibodies were detected with rabbit anti-mouse HRP antibody (1:20000 in blocking buffer without Na azide) for 1 h. After developing for 10 min with TMB substrate solution (Sigma-Aldrich Co.) and stopping the reaction with 1 m H_2_SO_4_, HRP activity was measured in a plate reader (Molecular Devices, Thermo-max microplate reader) at 450 nm.

### Western blot analysis

Cells were collected by trypsinization and pelleted at 700 g for 10 min at room temperature. The pellet was lysed in 50–100 μl of lysis buffer (10 mm Tris, 150 mm NaCl, pH 7.4, 1% v/v Nonidet P40, 1 mm PMSF, protease inhibitor cocktail) and cleared by spinning at the highest speed in a bench centrifuge at 4°C for 10 min. The protein concentration was measured by a Bradford assay (Bio-Rad, Hemel Hempstead, UK). Fifty micrograms of protein was loaded in each lane and separated in 10% w/v SDS and 7.5% w/v non-denaturing gels and transferred on PVDF membrane as described in Ref. (43). Rabbit polyclonal anti-neuroserpin antibody was used at 1:10000 and secondary goat polyclonal anti-rabbit IgG (HRP) was used at 1:50000. The bands were visualized as described previously (43). Membranes were stripped in 0.2 N NaOH for 10 min and re-probed with a mouse monoclonal anti-actin antibody as a loading control.

### Confocal microscopy

Cells were grown and fixed for confocal microscopy, before staining for the ER and neuroserpin. To stain ER rabbit α calreticulin antibody was diluted to 1:500 (Thermo Scientific) and detected with Goat α-rabbit AF555 (Invitrogen) diluted 1:300. Neuroserpin staining was carried out with either the 1A10 antibody (to detect total neuroserpin) at 2.5 μg/ml or the 7C6 antibody (to detect neuroserpin polymers) at 3.5 μg/ml. Both 1A10 and 7C6 were detected with Goat α-mouse-AF488 (Invitrogen) diluted 1:300. Δ neuroserpin could not be stained with these antibodies as previously described (18). DNA was stained with Hoerscht stain at a 1:1000 dilution (Invitrogen).

### RNAi transfection

Cells were transfected using Oligofectamine (Invitrogen) at a final concentration of 40 nm in an antibiotic-free media. After 5 h, normal media was added to the cells. Cells were harvested 48 h after the transfection.

### Immunoprecipitation and ubiquitin western blot analysis

Cells were treated and induced as described previously. To detect ubiquitin after immunoprecipitaiton, cells were transfected with wild-type ubiquitin (in pcDNA3.1) 48 h before the experiment. During the last 4 h, cells were treated with MG132 40 µm to inhibit proteasomal degradation. Cells were harvested like described previously and lysed in the same lysis buffer supplemented with 10 mm IAA (Sigma). Cell lysate samples were pre-cleared with rabbit IgG bound to 45 μl of 50% (v/v) Protein A-Sepharose for 30 min at 4°C, and then neuroserpin was immunoprecipitated overnight at 4°C with specific antibodies pre-bound to Protein A-Sepharose (45 μl of 50% (v/v) Protein A-Sepharose plus 1 μg of purified antibody at 4°C for 2 h). The following day, immunocomplexes were washed four times with cold PBS and once with washing buffer (150 mm NaCl, 50 mm Tris-Cl, pH 7.5, 1% (v/v) Nonidet P-40). Immunocomplex proteins were recovered in SDS–PAGE loading buffer by heating for 5 min at 90°C, separated on 4–12% w/v Bis-Tris Nupage gel (Invitrogen) and detected by western blot analysis using the P4D1 antibody (Santa Cruz) against ubiquitin.

### Microarray and analysis

The Cambridge Genomic Service (http://www.cgs.path.cam.ac.uk) performed the microarray experiments and analysis. Briefly, total RNA from HeLa Wt, G392E and Δ cells was collected using Trizol (Sigma). RNA amplification was performed using Ambion Total Prep kit (Ambion, UK) and microarrays were undertaken using Illumina chips according to the manufacturer's protocol. Raw data were pre-processed using R software (http://www.r-project.org) and the bioconductor (http://www.bioconductor.org) package lumi (45). Probes were removed from the data set where the Illumina detection *P*-value was greater than 0.01. The data were then transformed using the variance stabilization transformation algorithm (VST) and normalized using the quantile normalization method. Differentially expressed genes for each condition (wild-type neuroserpin ‘on’ versus wild-type neuroserpin ‘off’, G392E neuroserpin ‘on’ versus G392E neuroserpin ‘off’ and Δ neuroserpin ‘on’ versus Δ neuroserpin ‘off’’) were identified by fitting linear models with the bioconductor R package limma (46). To account for multiple hypotheses testing, *P*-values were adjusted using the Benjamini & Hochberg false discovery rate (FDR) correction. In order to assess the gene expression profiles at a pathway level, Gene Set Enrichment analysis (27) was performed on the normalized gene expression values for each group. Gene Set Enrichment analysis uses *a priori* defined gene sets to determine pathway differences between two phenotypic states. Gene expression data are therefore evaluated at the pathway level rather than the single gene. Gene Set Enrichment analysis first ranks the genes according to their relative difference in gene expression. The ranked list is then compared with gene sets (pathways) and an enrichment score (ES) is calculated for each gene. When a gene is present in the gene set of interest, the running ES is increased. If the gene is absent the running ES is decreased. The enrichment statistic is the maximum deviation of the running ES from zero. Statistical significance is assessed by performing a permutation test procedure. To account for the differences in gene set size, a normalized ES is calculated. Estimated significance levels are also adjusted for multiple hypothesis testing. FDR is the estimated probability that a gene set with a given enrichment statistic represents a false-positive finding. A total of 639 pathways (gene sets) were analysed from Kyoto Encyclopedia of Genes and Genomes, Reactome and BioCarta databases (http://www.genome.jp/kegg/pathway.html, http://www.reactome.org/ and http://www.biocarta.com/genes/index.asp, respectively). Gene sets containing less than 15 and greater than 500 genes were filtered out, along with gene sets containing no genes from the input data set.

### Luciferase assay

To measure SREBP2 activity in HeLa cells, a luciferase reporter plasmid was created using a design described previously (47). The promoter region of *Cricetulus griseus* HMG synthase from −368 to +1 bp, containing SREBP binding sites, was constructed by Eurofins (Germany) with *Sma*I and *Xho*I restriction sites. This region was cloned into the luciferase reporter pGL2 basic vector (Promega, UK). Cells were transfected with 1 μg of pGL2–HMG synthase and 50 ng of the control plasmid pRL-TK (Promega) 72 h before collection. Treatment with either 2 μg/ml doxycycline or 5 μm simvastatin was administered 48 h before collection. Measurement of pGL2-HMG synthase relative to pRL-TK was carried out with the Dual–Luciferase Reporter Assay System (Promega).

### Statistical analysis

All graphics represent mean ± SEM. Mann–Whitney analysis was performed in all our experiments unless otherwise mentioned. *P < 0.05 and **P < 0.01.

## SUPPLEMENTARY MATERIAL

Supplementary Material is available at *HMG* online.

*Conflict of Interest statement*. The authors have no conflicts of interest.

## FUNDING

This work was funded by the Medical Research Council (UK), the Engineering and Physical Sciences Council and GlaxoSmithKline. S.J.M. is an MRC Senior Clinical Fellow, P.J.L. is a Wellcome Trust Senior Clinical Fellow and M.L.B. is a Cambridge NIHR BRC Clinical Training Fellow.

## Supplementary Material

Supplementary Data
