# Supplementary material for: Sterol metabolism regulates neuroserpin polymer degradation in the absence of the unfolded protein response in the dementia FENIB
Source: Hum Mol Genet. 2013 Jun 28;22(22):4616–26. doi: 10.1093/hmg/ddt310 (PMC3889810; doi:10.1093/hmg/ddt310)
Supplement: Supplementary Data [file supp_22_22_4616__index.html]

Sterol metabolism regulates neuroserpin polymer degradation in the absence of the unfolded protein response in the dementia FENIB — Sterol metabolism regulates neuroserpin polymer degradation in the absence of the unfolded protein response in the dementia FENIB — Supplementary Data 

# Sterol metabolism regulates neuroserpin polymer degradation in the absence of the unfolded protein response in the dementia FENIB

## 

Supplementary Data

**Files in this Data Supplement:**

- Supplementary Data - Pdf file
