# Supplementary material for: Sterol metabolism regulates neuroserpin polymer degradation in the absence of the unfolded protein response in the dementia FENIB
Source: Hum Mol Genet. 2013 Jun 28;22(22):4616–26. doi: 10.1093/hmg/ddt310 (PMC3889810; doi:10.1093/hmg/ddt310)
Supplement: Supplementary Data [file supp_ddt310_ddt310supp.pdf]

Supplementary material:

Gene expression analysis

Total RNA was isolated using Tri-Reagent (Sigma-Aldrich) according to the manufacturer's instructions. Contaminating genomic DNA was removed by DNAseI treatment with Turbo DNA free (Applied Biosystems/Ambion). Total RNA (100 ng) was used for cDNA synthesis, using random primers and MMLV reverse transcriptase (Invitrogen). Human HMGCoA reductase, human HMGCoA synthase, human MVD, human LSS and human actin mRNA levels were measured by real-time PCR using the primer sets listed in Supplementary Table 4. Relative expression was calculated as a ratio to the actin signal.

Fly analysis

All used fly stocks were in a w^{1118} background and maintained on standard fly food with the addition of dried yeast at 25°C. We used transgenic *Drosophila* lines expressing wild type and G392E neuroserpin as described previously (18). Both lines contain the human secretion signal peptide sequence but also proline substitutions at positions P1 and P1' of the reactive loop to abolish the intrinsic protease inhibitory activity that has been shown to cause toxicity in *Drosophila* (46). This substitution does not to alter the polymerisation properties of mutant neuroserpin (18). The Gal4/UAS system was used for ectopic gene expression in *Drosophila* (47). Neuroserpin was expressed in neuronal cells with the elav-Gal4 driver. UAS-RNAi lines against the *Drosophila* homologues of the E2 and E3 ligases were obtained from the Vienna Drosophila RNAi center (Vienna, Austria). Only one *Drosophila*

homologue was found to represent the mammalian genes UBE2J1 and UBE2J2 (CG5823) with CG1937 being the homologue of Hrd1 and Gp78. The *Drosophila* gene CG4443 encodes the mammalian homologue UBE2G2. Stable Drosophila lines expressing neuroserpin in the nervous system driven by elav-Gal4 were generated by the use of the second and third chromosome balancers, CurlyO and TM6b,tb respectively. Female virgin flies were then crossed with males of each RNAi line. All crosses were performed at 25°C. Progeny flies expressing neuroserpin and the RNAi construct were collected at the day of eclosion and aged for 35 days at 25°C by changing the food every second/third day. After 35 days flies were collected and frozen at -80°C until further processing. The following VDRC RNAi lines were used in this experiment: RNAi against: CG1937: v6870(GD),v107060 (KK); CG5823: v8301(GD), v108292 (KK); CG4443: v34111 (GD), v104440 (KK); CG8711: v44829 (GD), v105668 (KK); CG7425: v26011 (GD), v105731 (KK).

Supplementary Figure 1. Depletion of CG1937 (E3) or CG4443 (E2) ligases increases the level of neuroserpin in a *Drosophila melanogaster* model of FENIB. Levels of total neuroserpin were determined in transgenic fly brain homogenates by ELISA directed against total neuroserpin, in flies expressing Wt (**A**) or G392E (**B**) neuroserpin. Flies were crossed with UAS-RNAi lines against both E3 ligases hrd1 and gp78 (CG1937), both E2 ligases UBE2j1 and UBE2j2 (CG5823) or the E2 ligase UBE2g2 (CG4443). A minimum of 100 progeny were analysed per each cross. The animals were collected in pools of minimum of 25 animals from multiple vials from each cross set up under the same conditions. Histograms represent mean and standard deviation. *: $p<0.05$.

Supplementary Figure 2. Microarray analysis of cells expressing neuroserpin and its mutants. (**A**) Heat map of average expression values for each group for the top 100 genes with the greatest variance between groups. Multiple probes were collapsed to one gene by selecting the probe with highest average expression. Each group is represented by a column and each gene is represented by a row. The dendrogram depicts hierarchical clustering based on the gene expression values. Scaled expression values are colour coded as represented by the legend on the left. List of genes with a logFC above or below 0.5 in wild type cells(**B**), G392E cells (**C**) and in Delta neuroserpin cells (**D**).

Supplementary Figure 3. RT-qPCR analysis of the cholestertol biosynthesis pathway in HeLa cell lines that conditionally express wild-type and mutant neuroserpin. Expression of HMGCoA reductase (**A**), HMGCoA synthase (**B**), mevalonate (diphospho) decarboxylase (MVD) (**C**), and lanosterol synthase (LSS)

(**D**) normalized to actin in HeLa cells expressing wild-type, G392E or Δ neuroserpin. The cells were treated with 2μg/mL doxycycline for 48 hours and the data are the mean +/- SEM of 3 independent experiments. *: $p<0.05$.

Supplementary Table 1. Upregulated pathways when wild-type neuroserpin is expressed in HeLa cells. Table of the 54 upregulated pathways in wild-type cells following treatment with 2μg/mL doxycycline. Analysis of the microarray was performed as described in the material and methods section. The 54 pathways are filtered according a cut off of FDR <=0.25. NAME: pathway name, ES: enrichment score, NES: normalized enrichment score, NOM p-val: the nominal p value estimates the statistical significance of the enrichment score for a single gene set, FDR q-val: False discovery rate; that is, the estimated probability that the normalized enrichment score represents a false positive finding.

Supplementary Table 2. Upregulated pathways when G392E neuroserpin is expressed in HeLa cells. Table of the 70 upregulated pathways in G392E cells following treatment with doxycycline. Analysis of the microarray was performed as described in the material and methods section. The 70 pathways are filtered according a cut off of FDR <=0.25. NAME: pathway name, ES: enrichment score, NES: normalized enrichment score, NOM p-val: the nominal p value estimates the statistical significance of the enrichment score for a single gene set, FDR q-val: False discovery rate; that is, the estimated probability that the normalized enrichment score represents a false positive finding.

Supplementary Table 3. RT-qPCR primers. Table of the forward and reverse primer sequences used in our qPCR experiments.

Sup Figure 1

A

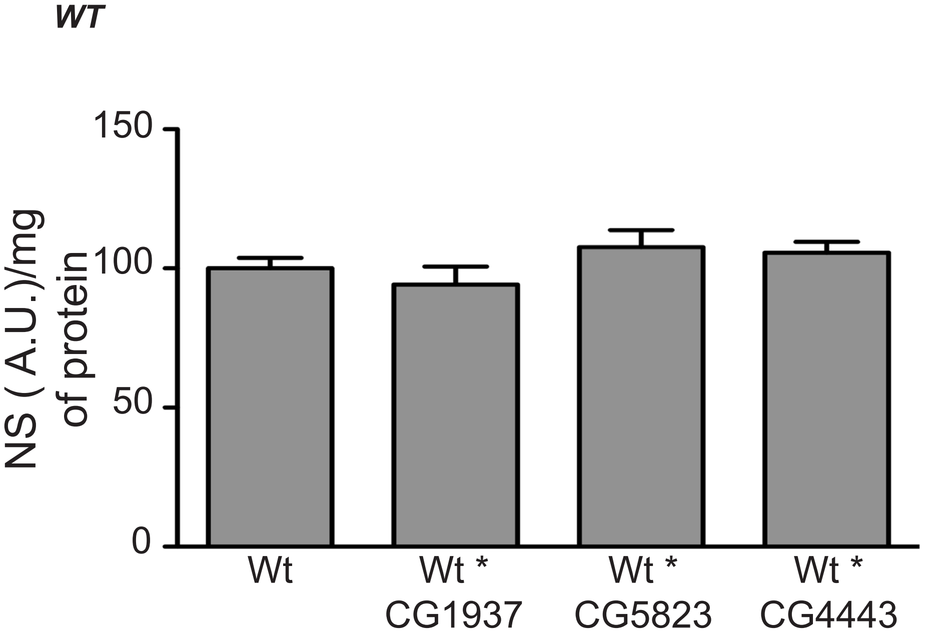

B

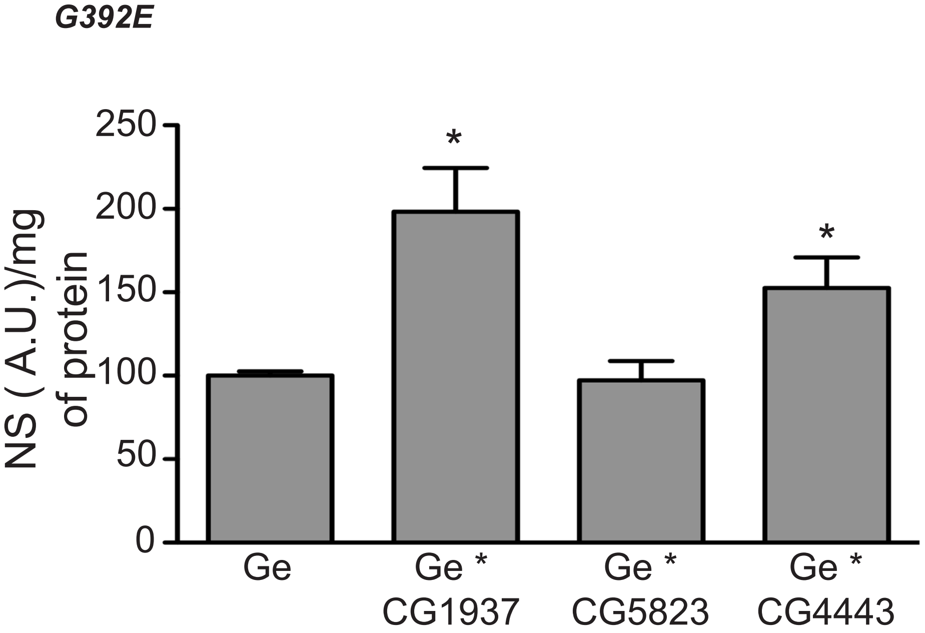

Sup Figure 2

A

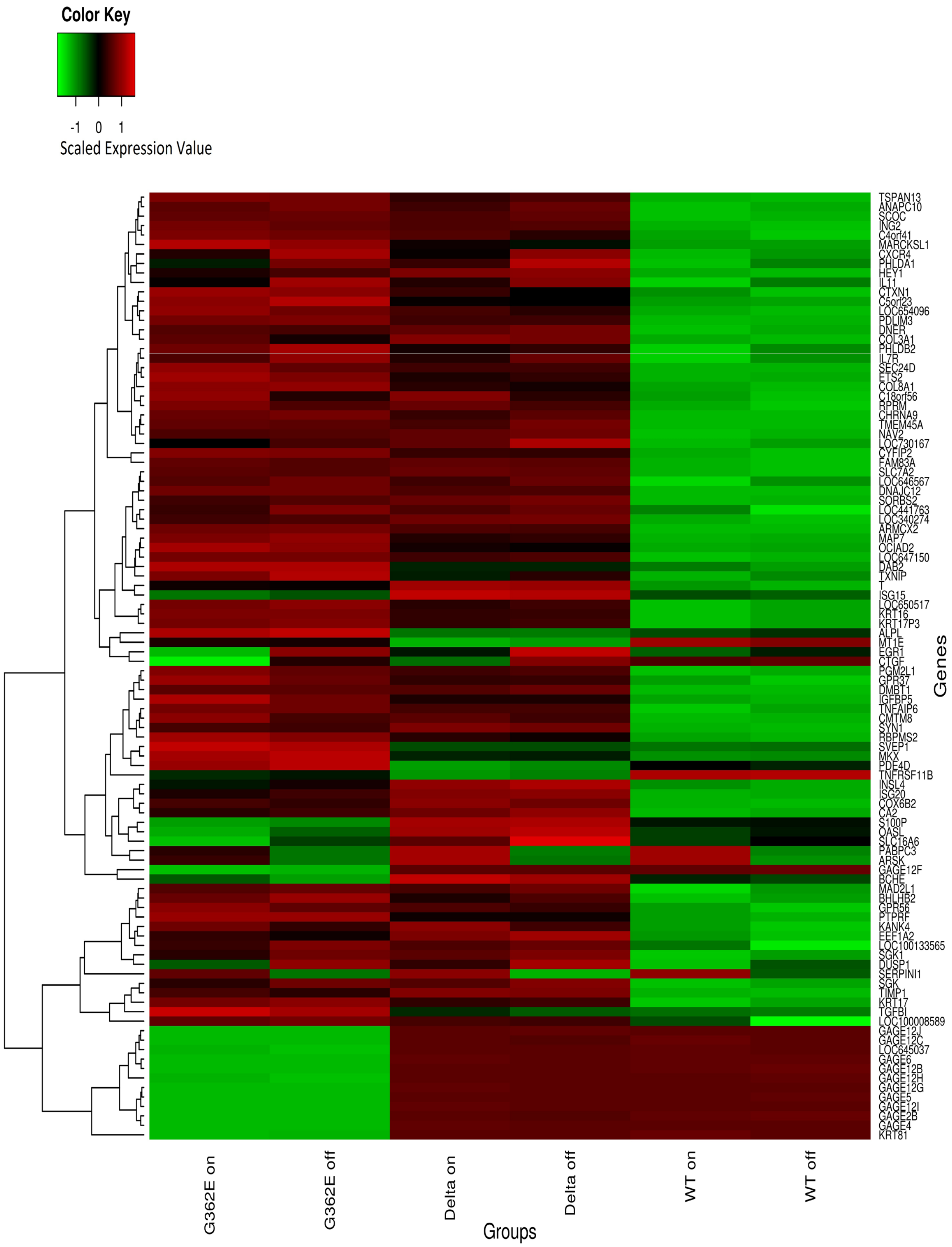

B

SYMBOL	DEFINITION	logFC	P.Value
SERPINI1	Homo sapiens serpin peptidase inhibitor, clade I (neuroserpin), member 1 (SERPINI1), mRNA.	3,78224072	1,05E-08
SERPINI1	Homo sapiens serpin peptidase inhibitor, clade I (neuroserpin), member 1 (SERPINI1), mRNA.	3,7320891	2,56E-07
ARSK	Homo sapiens arylsulfatase K (ARSK), mRNA.	2,27785895	7,10E-06
PABPC3	Homo sapiens poly(A) binding protein, cytoplasmic 3 (PABPC3), mRNA.	1,54307413	4,44E-05
ZP3	Homo sapiens zona pellucida glycoprotein 3 (sperm receptor) (ZP3), mRNA.	1,52877557	3,10E-05
C16orf53	Homo sapiens chromosome 16 open reading frame 53 (C16orf53), mRNA.	1,31553055	3,89E-06
SNAI2	Homo sapiens snail homolog 2 (Drosophila) (SNAI2), mRNA.	0,91798125	0,00382311
PRNPIP	PREDICTED: Homo sapiens prion protein interacting protein, transcript variant 2 (PRNPIP), mRNA.	0,78825948	0,00161071
MBLAC2	Homo sapiens metallo-beta-lactamase domain containing 2 (MBLAC2), mRNA.	0,70124753	0,00106466
SLC12A3	Homo sapiens solute carrier family 12 (sodium/chloride transporters), member 3 (SLC12A3), mRNA.	0,69647612	0,08327948
HAGHL	Homo sapiens hydroxyacylglutathione hydrolase-like (HAGHL), transcript variant 2, mRNA.	0,69174877	0,00264439
CROT	Homo sapiens carnitine O-octanoyltransferase (CROT), mRNA.	0,65652441	0,03891727
FLJ20444	PREDICTED: Homo sapiens hypothetical protein FLJ20444, transcript variant 2 (FLJ20444), mRNA.	0,63716557	0,00095198
LOC728936	PREDICTED: Homo sapiens similar to CG10522-PA, transcript variant 6 (LOC728936), mRNA.	0,61246809	0,00111891
OR4K13	Homo sapiens olfactory receptor, family 4, subfamily K, member 13 (OR4K13), mRNA.	0,61001045	0,00090371
VAT1	Homo sapiens vesicle amine transport protein 1 homolog (T. californica) (VAT1), mRNA.	0,60104646	0,02038369
H19	Homo sapiens H19, imprinted maternally expressed transcript (non-protein coding) (H19), non-coding RNA.	0,59018298	0,01823104
YWHAE	Homo sapiens tyrosine 3-monooxygenase/tryptophan 5-monooxygenase activation protein, epsilon polypeptide (YWHAE), mRNA.	0,58331983	0,09924143
RABGAP1	Homo sapiens RAB GTPase activating protein 1 (RABGAP1), mRNA.	0,57161758	0,02885626
IL11	Homo sapiens interleukin 11 (IL11), mRNA.	-1,1053049	0,01710589
LOC652233	PREDICTED: Homo sapiens hypothetical protein LOC652233 (LOC652233), mRNA.	-0,9249651	0,102521
KRT17	Homo sapiens keratin 17 (KRT17), mRNA.	-0,9235621	0,1168352
TSC22D1	Homo sapiens TSC22 domain family, member 1 (TSC22D1), transcript variant 2, mRNA.	-0,9195618	0,11826578
ATF3	Homo sapiens activating transcription factor 3 (ATF3), transcript variant 4, mRNA.	-0,9083858	0,00314757
CCNC	Homo sapiens cyclin C (CCNC), transcript variant 1, mRNA.	-0,871588	0,16222196
SC4MOL	Homo sapiens sterol-C4-methyl oxidase-like (SC4MOL), transcript variant 1, mRNA.	-0,8345705	0,18404266
HAT1	Homo sapiens histone acetyltransferase 1 (HAT1), transcript variant 1, mRNA.	-0,8136781	0,07433243
MEST	Homo sapiens mesoderm specific transcript homolog (mouse) (MEST), transcript variant 2, mRNA.	-0,8071275	0,17095893
EIF4G2	Homo sapiens eukaryotic translation initiation factor 4 gamma, 2 (EIF4G2), transcript variant 1, mRNA.	-0,8025218	0,11080876
CXCR4	Homo sapiens chemokine (C-X-C motif) receptor 4 (CXCR4), transcript variant 2, mRNA.	-0,7778527	0,28001737
TMPO	Homo sapiens thymopoietin (TMPO), transcript variant 1, mRNA.	-0,7777228	0,10901463
KRT17P3	PREDICTED: Homo sapiens misc_RNA (KRT17P3), miscRNA.	-0,771574	0,14468966
DUSP1	Homo sapiens dual specificity phosphatase 1 (DUSP1), mRNA.	-0,764261	0,15679331
LOC644063	PREDICTED: Homo sapiens similar to heterogeneous nuclear ribonucleoprotein K (LOC644063), mRNA.	-0,7551716	0,16932469
BZW1	Homo sapiens basic leucine zipper and W2 domains 1 (BZW1), mRNA. XM_943165	-0,7387113	0,09315804
LOC730029	PREDICTED: Homo sapiens similar to hCG1997137, transcript variant 2 (LOC730029), mRNA.	-0,7248607	0,06039956
LOC10012841	PREDICTED: Homo sapiens misc_RNA (LOC100128410), miscRNA.	-0,720418	0,00884764
MRPL39	Homo sapiens mitochondrial ribosomal protein L39 (MRPL39), nuclear gene encoding mitochondrial protein, transcript variant 1, m	-0,7144454	0,02150847
LOC650517	PREDICTED: Homo sapiens hypothetical LOC650517 (LOC650517), mRNA.	-0,7058884	0,1739138
TCP1	Homo sapiens t-complex 1 (TCP1), transcript variant 1, mRNA.	-0,7042835	0,16860849
RSPO3	Homo sapiens R-spondin 3 homolog (Xenopus laevis) (RSPO3), mRNA.	-0,6979643	0,00314593
SLC38A1	Homo sapiens solute carrier family 38, member 1 (SLC38A1), transcript variant 1, mRNA.	-0,6886323	0,18355396
TWF1	Homo sapiens twinfilin, actin-binding protein, homolog 1 (Drosophila) (TWF1), mRNA.	-0,6884723	0,17014969
C8orf4	Homo sapiens chromosome 8 open reading frame 4 (C8orf4), mRNA.	-0,6866275	0,09648224
LOC728059	PREDICTED: Homo sapiens misc_RNA (LOC728059), miscRNA.	-0,6829107	0,22776352
	AV737317 CB Homo sapiens cDNA clone CBCAQH03 5, mRNA sequence	-0,6796887	0,24137685
IL6	Homo sapiens interleukin 6 (interferon, beta 2) (IL6), mRNA.	-0,6693901	0,07364386
MRPL47	Homo sapiens mitochondrial ribosomal protein L47 (MRPL47), nuclear gene encoding mitochondrial protein, transcript variant 1, m	-0,6648882	0,00873302
TCEA1	Homo sapiens transcription elongation factor A (SII), 1 (TCEA1), transcript variant 2, mRNA.	-0,6535575	0,24469875
EFEMP1	Homo sapiens EGF-containing fibulin-like extracellular matrix protein 1 (EFEMP1), transcript variant 1, mRNA.	-0,6528274	0,23373945
LOC648695	PREDICTED: Homo sapiens similar to retinoblastoma binding protein 4, transcript variant 5 (LOC648695), mRNA.	-0,6445514	0,10617075
GNG10	Homo sapiens guanine nucleotide binding protein (G protein), gamma 10 (GNG10), mRNA.	-0,6293811	0,05011279
KRT16	Homo sapiens keratin 16 (focal non-epidermolytic palmoplantar keratoderma) (KRT16), mRNA.	-0,6292386	0,17336413
BMP6	Homo sapiens bone morphogenetic protein 6 (BMP6), mRNA.	-0,6267273	0,13505334
CXCR4	Homo sapiens chemokine (C-X-C motif) receptor 4 (CXCR4), transcript variant 1, mRNA.	-0,6255164	0,05399763
SLC39A6	Homo sapiens solute carrier family 39 (zinc transporter), member 6 (SLC39A6), mRNA.	-0,6199442	0,01897618
LOC10012792	PREDICTED: Homo sapiens misc_RNA (LOC100127922), miscRNA.	-0,617164	0,00457621
TWF1	Homo sapiens twinfilin, actin-binding protein, homolog 1 (Drosophila) (TWF1), mRNA.	-0,6149413	0,17890799
TCEA1	Homo sapiens transcription elongation factor A (SII), 1 (TCEA1), transcript variant 1, mRNA.	-0,6124938	0,09365265
TAF9	Homo sapiens TAF9 RNA polymerase II, TATA box binding protein (TBP)-associated factor, 32kDa (TAF9), transcript variant 2, mR	-0,6111431	0,11571637
ACN9	Homo sapiens ACN9 homolog (S. cerevisiae) (ACN9), mRNA.	-0,6105095	0,14710544
LOC730167	PREDICTED: Homo sapiens similar to protein tyrosine phosphatase 4a1, transcript variant 1 (LOC730167), mRNA.	-0,6063494	0,1983058
RRM2	Homo sapiens ribonucleotide reductase M2 polypeptide (RRM2), mRNA.	-0,5987869	0,06930307
SLC25A24	Homo sapiens solute carrier family 25 (mitochondrial carrier; phosphate carrier), member 24 (SLC25A24), nuclear gene encoding	-0,5985318	0,22803299
PREI3	Homo sapiens preimplantation protein 3 (PREI3), transcript variant 1, mRNA.	-0,5965332	0,04802725
CHAC2	Homo sapiens ChaC, cation transport regulator homolog 2 (E. coli) (CHAC2), mRNA.	-0,5948395	0,08118529
RFC3	Homo sapiens replication factor C (activator 1) 3, 38kDa (RFC3), transcript variant 1, mRNA.	-0,594123	0,11228466
LOC10013383	PREDICTED: Homo sapiens hypothetical protein LOC100133836, transcript variant 1 (LOC100133836), mRNA.	-0,5939306	0,12392515
PRIM2A	PREDICTED: Homo sapiens primase, polypeptide 2A, 58kDa (PRIM2A), mRNA.	-0,586905	0,18301099
LOC643300	PREDICTED: Homo sapiens similar to 60 kDa heat shock protein, mitochondrial precursor (Hsp60) (60 kDa chaperonin) (CPN60) (	-0,5867672	0,09935211
LOC728188	PREDICTED: Homo sapiens similar to phosphoglycerate mutase processed protein (LOC728188), mRNA.	-0,5843347	0,141947
LOC10012908	PREDICTED: Homo sapiens similar to HIG1 domain family, member 1A (LOC100129086), mRNA.	-0,5822058	0,19487928
THOC3	Homo sapiens THO complex 3 (THOC3), mRNA.	-0,5802185	0,06714049
JUN	Homo sapiens jun oncogene (JUN), mRNA.	-0,5797673	0,11193186
SLC39A6	Homo sapiens solute carrier family 39 (zinc transporter), member 6 (SLC39A6), transcript variant 1, mRNA.	-0,5796835	0,17050742
PRDX3	Homo sapiens peroxiredoxin 3 (PRDX3), nuclear gene encoding mitochondrial protein, transcript variant 1, mRNA.	-0,5769827	0,21202081
LOC653566	Homo sapiens similar to Signal peptidase complex subunit 2 (Microsomal signal peptidase 25 kDa subunit) (SPase 25 kDa subunit)	-0,5752439	0,07455107
RCAN1	Homo sapiens regulator of calcineurin 1 (RCAN1), transcript variant 3, mRNA.	-0,5745296	0,13196616
C1orf124	Homo sapiens chromosome 1 open reading frame 124 (C1orf124), transcript variant 2, mRNA.	-0,5715377	0,0254855
CLEC2D	Homo sapiens C-type lectin domain family 2, member D (CLEC2D), transcript variant 1, mRNA.	-0,5705084	0,19873849
MORF4L2	Homo sapiens mortality factor 4 like 2 (MORF4L2), mRNA.	-0,5699261	0,17020417
MAT2B	Homo sapiens methionine adenosyltransferase II, beta (MAT2B), transcript variant 1, mRNA.	-0,5698141	0,12782482
G3BP2	Homo sapiens GTPase activating protein (SH3 domain) binding protein 2 (G3BP2), transcript variant 3, mRNA.	-0,5686238	0,10515
FASTKD3	Homo sapiens FAST kinase domains 3 (FASTKD3), mRNA.	-0,5677531	0,05017195
CNIH	Homo sapiens cornichon homolog (Drosophila) (CNIH), mRNA.	-0,5670736	0,15447516
LOC10013256	PREDICTED: Homo sapiens hypothetical protein LOC100132564 (LOC100132564), mRNA.	-0,5658825	0,07736043
MOCS2	Homo sapiens molybdenum cofactor synthesis 2 (MOCS2), transcript variant 1, mRNA.	-0,5656172	0,20016219
TAF9	Homo sapiens TAF9 RNA polymerase II, TATA box binding protein (TBP)-associated factor, 32kDa (TAF9), transcript variant 3, mR	-0,5621996	0,0493195
OLFM1	Homo sapiens olfactomedin 1 (OLFM1), transcript variant 2, mRNA.	-0,5603178	0,11517819
ZNF280C	Homo sapiens zinc finger protein 280C (ZNF280C), mRNA.	-0,5596996	0,00284224
LOC643873	PREDICTED: Homo sapiens misc_RNA (LOC643873), miscRNA.	-0,5582004	0,02885574
SMN1	Homo sapiens survival of motor neuron 1, telomeric (SMN1), transcript variant d, mRNA.	-0,5569524	0,07739212
ARL4A	Homo sapiens ADP-ribosylation factor-like 4A (ARL4A), transcript variant 1, mRNA.	-0,5564705	0,02219619
LOC729686	PREDICTED: Homo sapiens misc_RNA (LOC729686), miscRNA.	-0,5545166	0,0053888
LOC649555	PREDICTED: Homo sapiens similar to eukaryotic translation initiation factor 4E, transcript variant 2 (LOC649555), mRNA.	-0,5538529	0,07036284
CCT6A	Homo sapiens chaperonin containing TCP1, subunit 6A (zeta 1) (CCT6A), transcript variant 1, mRNA.	-0,5537792	0,16970749
SEH1L	Homo sapiens SEH1-like (S. cerevisiae) (SEH1L), transcript variant 1, mRNA.	-0,5522498	0,01210908
CENPK	Homo sapiens centromere protein K (CENPK), mRNA.	-0,5507598	0,14239611

C

SYMBOL	DEFINITION	logFC	P.Value
SERPINI1	Homo sapiens serpin peptidase inhibitor, clade I (neuroserpin), member 1 (SERPINI1), mRNA.	3,53392644	2,68E-10
SERPINI1	Homo sapiens serpin peptidase inhibitor, clade I (neuroserpin), member 1 (SERPINI1), mRNA.	3,52937076	7,36E-10
RNU4-1	Homo sapiens RNA, U4 small nuclear 1 (RNU4-1), small nuclear RNA.	1,32359926	0,00029918
ARSK	Homo sapiens arylsulfatase K (ARSK), mRNA.	1,28104555	3,07E-06
RNU11	Homo sapiens RNA, U11 small nuclear (RNU11), small nuclear RNA.	1,17154731	0,00310001
	BP873537 Sugano cDNA library, embryonal kidney Homo sapiens cDNA clone HKR13896, mRNA sequence	0,99396645	0,00075571
RNU4-2	Homo sapiens RNA, U4 small nuclear 2 (RNU4-2), small nuclear RNA.	0,98754431	6,18E-05
TERC	Homo sapiens telomerase RNA component (TERC), telomerase RNA.	0,96108739	0,00095042
PABPC3	Homo sapiens poly(A) binding protein, cytoplasmic 3 (PABPC3), mRNA.	0,87688878	0,00027069
C16orf53	Homo sapiens chromosome 16 open reading frame 53 (C16orf53), mRNA.	0,81812854	0,00027143
	Human small nuclear RNA U6atac, partial sequence	0,76912275	0,01986007
ZP3	Homo sapiens zona pellucida glycoprotein 3 (sperm receptor) (ZP3), mRNA.	0,73937574	5,17E-05
RNU6ATAC	Homo sapiens RNA, U6atac small nuclear (U12-dependent splicing) (RNU6ATAC), small nuclear RNA.	0,73449589	0,01854801
C14orf78	PREDICTED: Homo sapiens chromosome 14 open reading frame 78 (C14orf78), mRNA.	0,70390305	0,00104648
LOC1001298	PREDICTED: Homo sapiens similar to mCG49427 (LOC100129882), mRNA.	0,65613153	0,0005387
MT1F	Homo sapiens metallothionein 1F (MT1F), mRNA.	0,65339329	0,00031199
AHNAK2	Homo sapiens AHNAK nucleoprotein 2 (AHNAK2), mRNA.	0,64291493	0,00107838
DIDO1	Homo sapiens death inducer-obliterator 1 (DIDO1), transcript variant 3, mRNA.	0,63348524	0,00123956
RNU1-3	Homo sapiens RNA, U1 small nuclear 3 (RNU1-3), small nuclear RNA.	0,62099329	0,00021775
SNORA12	Homo sapiens small nucleolar RNA, H/ACA box 12 (SNORA12), small nucleolar RNA.	0,6027284	0,00290094
MXD3	Homo sapiens MAX dimerization protein 3 (MXD3), mRNA.	0,59542452	0,00012898
RNU1-5	Homo sapiens RNA, U1 small nuclear 5 (RNU1-5), small nuclear RNA.	0,59015128	0,00241739
VAV3	Homo sapiens vav 3 guanine nucleotide exchange factor (VAV3), transcript variant 1, mRNA.	0,57219344	0,00045121
ACSF2	Homo sapiens acyl-CoA synthetase family member 2 (ACSF2), mRNA.	0,55619369	0,00118382
RDM1	Homo sapiens RAD52 motif 1 (RDM1), transcript variant 2, mRNA.	0,55157402	0,01245924
EGR1	Homo sapiens early growth response 1 (EGR1), mRNA.	-1,7482123	0,00041997
FOS	Homo sapiens v-fos FBJ murine osteosarcoma viral oncogene homolog (FOS), mRNA.	-1,1893669	0,00247487
CTGF	Homo sapiens connective tissue growth factor (CTGF), mRNA.	-1,1788248	0,00031087
DUSP1	Homo sapiens dual specificity phosphatase 1 (DUSP1), mRNA.	-1,0208394	0,00024636
IL11	Homo sapiens interleukin 11 (IL11), mRNA.	-0,9768607	1,34E-05
CYR61	Homo sapiens cysteine-rich, angiogenic inducer, 61 (CYR61), mRNA.	-0,8982975	0,00169414
IL6	Homo sapiens interleukin 6 (interferon, beta 2) (IL6), mRNA.	-0,8792323	2,91E-05
PTGS2	Homo sapiens prostaglandin-endoperoxide synthase 2 (prostaglandin G/H synthase and cyclooxygenase) (PTGS2	-0,8627838	0,00108433
CTGF	Homo sapiens connective tissue growth factor (CTGF), mRNA.	-0,8308677	0,00077321
ATF3	Homo sapiens activating transcription factor 3 (ATF3), transcript variant 4, mRNA.	-0,791728	8,00E-05
PTGS2	Homo sapiens prostaglandin-endoperoxide synthase 2 (prostaglandin G/H synthase and cyclooxygenase) (PTGS2	-0,7261511	0,00018915
MIR21	Homo sapiens microRNA 21 (MIR21), microRNA.	-0,6723387	0,00269544
JUN	Homo sapiens jun oncogene (JUN), mRNA.	-0,6722707	0,00145817
FOXQ1	Homo sapiens forkhead box Q1 (FOXQ1), mRNA.	-0,6692236	0,00680116
PHLDA1	Homo sapiens pleckstrin homology-like domain, family A, member 1 (PHLDA1), mRNA.	-0,6656224	0,03983631
ODC1	Homo sapiens ornithine decarboxylase 1 (ODC1), mRNA.	-0,6093603	0,00761732
LOC644422	PREDICTED: Homo sapiens misc_RNA (LOC644422), miscRNA.	-0,6057286	0,00044824
CXCR4	Homo sapiens chemokine (C-X-C motif) receptor 4 (CXCR4), transcript variant 2, mRNA.	-0,6043377	0,12262504
SNAI2	Homo sapiens snail homolog 2 (Drosophila) (SNAI2), mRNA.	-0,5925466	0,00356245
	AGENCOURT_10229596 NIH_MGC_141 Homo sapiens cDNA clone IMAGE:6563923 5, mRNA sequence	-0,5801955	0,22755016
SLC16A6	Homo sapiens solute carrier family 16, member 6 (monocarboxylic acid transporter 7) (SLC16A6), mRNA.	-0,574914	0,00195379
SGK1	Homo sapiens serum/glucocorticoid regulated kinase 1 (SGK1), transcript variant 1, mRNA.	-0,5709317	0,03346349

D

SYMBOL	DEFINITION	logFC	P.Value
SERPINI1	Homo sapiens serpin peptidase inhibitor, clade I (neuroserpin), member 1 (SERPINI1), mRNA.	5,23532458	1,10E-13
ARSK	Homo sapiens arylsulfatase K (ARSK), mRNA.	2,05778431	9,76E-09
PABPC3	Homo sapiens poly(A) binding protein, cytoplasmic 3 (PABPC3), mRNA.	1,56342864	1,61E-07
ZP3	Homo sapiens zona pellucida glycoprotein 3 (sperm receptor) (ZP3), mRNA.	1,48693508	3,26E-08
C16orf53	Homo sapiens chromosome 16 open reading frame 53 (C16orf53), mRNA.	1,23631712	1,07E-06
SNAI2	Homo sapiens snail homolog 2 (Drosophila) (SNAI2), mRNA.	0,82946091	5,49E-06
LOC728936	PREDICTED: Homo sapiens similar to CG10522-PA, transcript variant 6 (LOC728936), mRNA.	0,78153333	2,32E-05
HAGHL	Homo sapiens hydroxyacylglutathione hydrolase-like (HAGHL), transcript variant 2, mRNA.	0,68456294	8,56E-06
CRELD2	Homo sapiens cysteine-rich with EGF-like domains 2 (CRELD2), mRNA.	0,55773887	0,00225945
C14orf78	PREDICTED: Homo sapiens chromosome 14 open reading frame 78 (C14orf78), mRNA.	0,55310315	0,00174484
EGR1	Homo sapiens early growth response 1 (EGR1), mRNA.	-1,1751655	0,00017127
CTGF	Homo sapiens connective tissue growth factor (CTGF), mRNA.	-1,0221491	0,00570959
CTGF	Homo sapiens connective tissue growth factor (CTGF), mRNA.	-0,9213526	0,00073677
IL6	Homo sapiens interleukin 6 (interferon, beta 2) (IL6), mRNA.	-0,8030559	0,00048287
FOS	Homo sapiens v-fos FBJ murine osteosarcoma viral oncogene homolog (FOS), mRNA.	-0,7684738	0,00090262
CYR61	Homo sapiens cysteine-rich, angiogenic inducer, 61 (CYR61), mRNA.	-0,7034378	0,0273799
IL11	Homo sapiens interleukin 11 (IL11), mRNA.	-0,6182015	0,00020606
CCNC	Homo sapiens cyclin C (CCNC), transcript variant 2, mRNA.	-0,6163162	0,04247062
PTGS2	Homo sapiens prostaglandin-endoperoxide synthase 2 (prostaglandin G/H synthase and cyclooxygenase) (PTGS2	-0,6044046	0,00475962
JUN	Homo sapiens jun oncogene (JUN), mRNA.	-0,603538	0,00086628
SLC16A6	Homo sapiens solute carrier family 16, member 6 (monocarboxylic acid transporter 7) (SLC16A6), mRNA.	-0,5988116	0,02247875
CXCR4	Homo sapiens chemokine (C-X-C motif) receptor 4 (CXCR4), transcript variant 2, mRNA.	-0,582671	0,09558186
TSC22D1	Homo sapiens TSC22 domain family, member 1 (TSC22D1), transcript variant 2, mRNA.	-0,5729566	0,04525485
PHLDA1	Homo sapiens pleckstrin homology-like domain, family A, member 1 (PHLDA1), mRNA.	-0,568877	0,02388658
NR4A2	Homo sapiens nuclear receptor subfamily 4, group A, member 2 (NR4A2), transcript variant 1, mRNA.	-0,5634467	0,00625389

Sup Figure 3

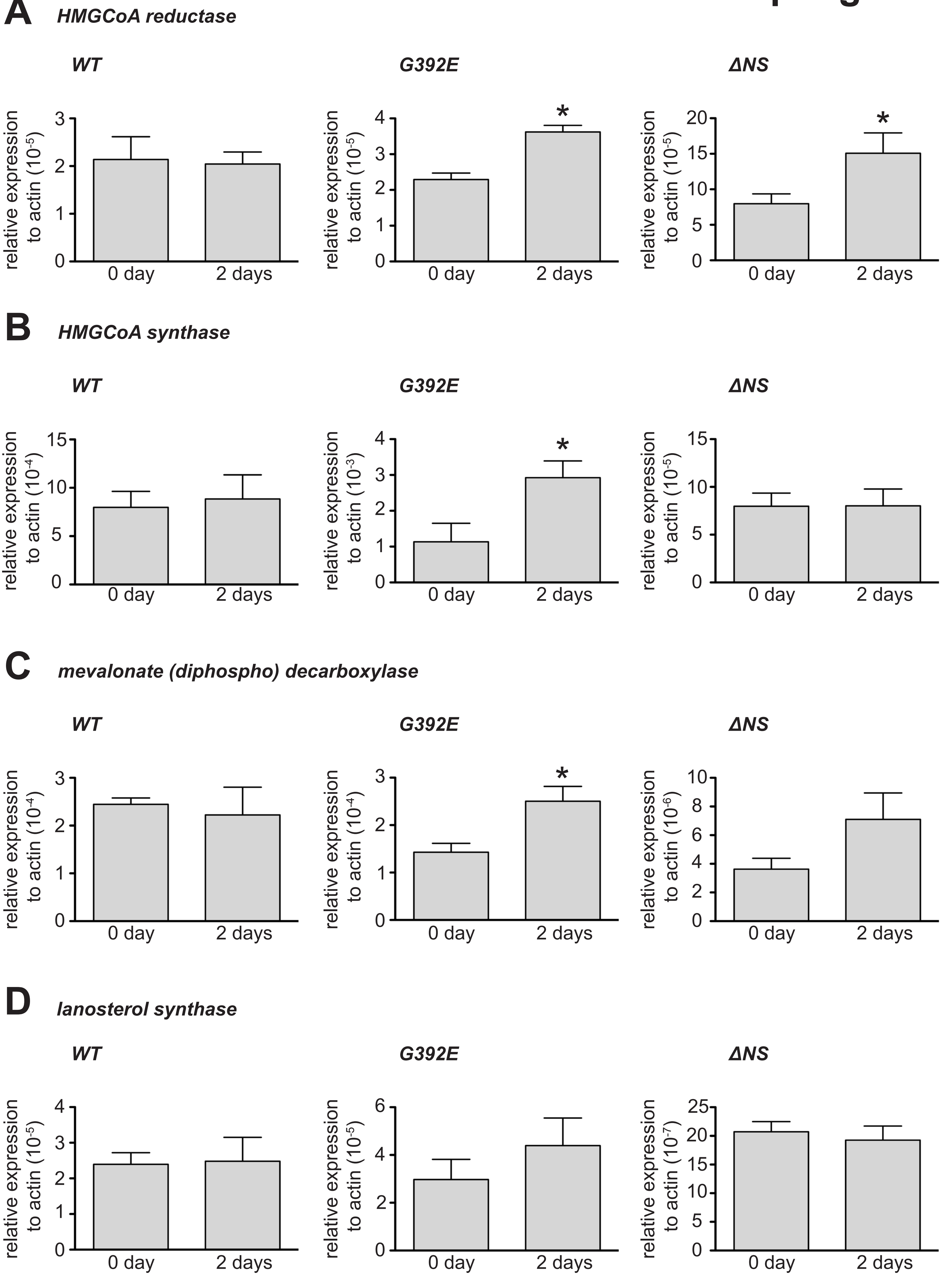
A
HMGCoA reductase
WT
G392E
ΔNS
relative expression to actin (10^{-5})
0 day
2 days
*
B
HMGCoA synthase
WT
G392E
ΔNS
relative expression to actin (10^{-4})
relative expression to actin (10^{-3})
relative expression to actin (10^{-5})
0 day
2 days
*
C
mevalonate (diphospho) decarboxylase
WT
G392E
ΔNS
relative expression to actin (10^{-4})
relative expression to actin (10^{-6})
0 day
2 days
*
D
lanosterol synthase
WT
G392E
ΔNS
relative expression to actin (10^{-5})
relative expression to actin (10^{-7})
0 day
2 days

NAME	ES	NES	NOM p-val	FDR q-val
HSA04910_INSULIN_SIGNALING_PATHWAY	0,47410455	2,0074332	0	0,20630583
HSA04150_MTOR_SIGNALING_PATHWAY	0,5689391	1,9857105	0	0,1265568
HSA04514_CELL_ADHESION_MOLECULES	0,50859135	1,9630845	0	0,10865378
HDACPATHWAY	0,6598234	1,9380826	0,005649718	0,102451466
PTENPATHWAY	0,66989946	1,8915575	0,005882353	0,11873982
HSA00260_GLYCINE_SERINE_AND_THREONINE_METABOLISM	0,58571386	1,8820919	0	0,10734854
HSA00071_FATTY_ACID_METABOLISM	0,5309787	1,8342218	0,007575758	0,13138427
HSA00620_PYRUVATE_METABOLISM	0,5210693	1,8204304	0,001798561	0,1267418
GLYCINE_SERINE_AND_THREONINE_METABOLISM	0,59576064	1,8057994	0,011650485	0,124381274
SIG_PIP3_SIGNALING_IN_B_LYMPHOCYTES	0,5622735	1,8025622	0,00984252	0,113860555
HSA00650_BUTANOATE_METABOLISM	0,531969	1,7464075	0,005747126	0,15357085
ST_PHOSPHOINOSITIDE_3_KINASE_PATHWAY	0,5463216	1,7317197	0,016363636	0,15640813
PROPANOATE_METABOLISM	0,544669	1,7254434	0,011627907	0,15017414
HSA04664_FC_EPSILON_RI_SIGNALING_PATHWAY	0,4615832	1,7030313	0,007476636	0,16080327
HSA00640_PROPANOATE_METABOLISM	0,5092561	1,6837213	0,015444015	0,17037958
ERKPATHWAY	0,51294833	1,6375355	0,021194605	0,21862705
HSA04670_LEUKOCYTE_TRANSENDOTHELIAL_MIGRATION	0,41575712	1,6336008	0,009633912	0,21196514
VALINE_LEUCINE_AND_ISOLEUCINE_DEGRADATION	0,4945509	1,6313512	0,013513514	0,20319831
HCMVPATHWAY	0,5778971	1,6170571	0,020872865	0,20959437
HSA00030_PENTOSE_PHOSPHATE_PATHWAY	0,51721126	1,6002023	0,02964427	0,22149795
IGF1MTORPATHWAY	0,53580004	1,5985835	0,028680688	0,21299642
ST_B_CELL_ANTIGEN_RECEPTOR	0,47268412	1,5906388	0,03018868	0,21432531
HSA04070_PHOSPHATIDYLINOSITOL_SIGNALING_SYSTEM	0,41813302	1,5878296	0,009025271	0,20831302
HSA01032_GLYCAN_STRUCTURES_DEGRADATION	0,49451143	1,580633	0,02247191	0,20931366
HSA00051_FRUCTOSE_AND_MANNOSE_METABOLISM	0,4592345	1,5801007	0,023076924	0,20165308
PYRUVATE_METABOLISM	0,4753586	1,5766352	0,026168223	0,19755904
ST_DICTYOSTELIUM_DISCOIDEUM_CAMP_CHEMOTAXIS_PATHWAY	0,4820396	1,5765147	0,032380953	0,19042937
INTEGRIN_MEDIATED_CELL_ADHESION_KEGG	0,39936882	1,5765141	0,01584507	0,18362832
HSA04650_NATURAL_KILLER_CELL_MEDIATED_CYTOTOXICITY	0,39673758	1,5755873	0,02268431	0,17847545
NDKDYNAMINPATHWAY	0,538975	1,5695479	0,033898305	0,17916106
HSA00280_VALINE_LEUCINE_AND_ISOLEUCINE_DEGRADATION	0,44038832	1,5582471	0,027726432	0,18592185
HSA04370_VEGF_SIGNALING_PATHWAY	0,40595648	1,5561677	0,017175572	0,1823264
BIOPEPTIDESPATHWAY	0,45165452	1,5523365	0,02631579	0,1813712
EIF4PATHWAY	0,50722593	1,5478889	0,0455408	0,180026
HSA00310_LYSINE_DEGRADATION	0,43169016	1,5410078	0,032490976	0,18274984
CREBPATHWAY	0,49766612	1,5288823	0,03358209	0,19056877
ST_ADRENERGIC	0,48771486	1,5218574	0,041516244	0,19378433
HSA00903_LIMONENE_AND_PINENE_DEGRADATION	0,4916303	1,5187186	0,036608864	0,19192621
HSA05214_GLIOMA	0,40599966	1,4989682	0,0375	0,21197678
HSA00010_GLYCOLYSIS_AND_GLUCONEOGENESIS	0,41404706	1,4957019	0,036072146	0,2107837
NKCELLSPATHWAY	0,51991475	1,4932169	0,044921875	0,20843992
MTORPATHWAY	0,48997015	1,491541	0,059760958	0,2055529
GLUCONEOGENESIS	0,4206949	1,4686764	0,060150377	0,23038498
GLYCOLYSIS	0,4206949	1,4617753	0,063097514	0,23367786
FRUCTOSE_AND_MANNOSE_METABOLISM	0,49432707	1,4611928	0,04990758	0,22913417
LYSINE_DEGRADATION	0,46063814	1,4587135	0,05065666	0,22775052
HSA00220_UREA_CYCLE_AND_METABOLISM_OF_AMINO_GROUPS	0,49452165	1,450584	0,055238094	0,23349902
ARGININE_AND_PROLINE_METABOLISM	0,44566956	1,4482433	0,057301294	0,2318463
PENTOSE_PHOSPHATE_PATHWAY	0,48559546	1,4449675	0,07777778	0,23106065
PHOSPHATIDYLINOSITOL_SIGNALING_SYSTEM	0,36149302	1,442951	0,0503876	0,2290126
FMLPPATHWAY	0,43700874	1,4404056	0,053846154	0,22754197
SIG_BCR_SIGNALING_PATHWAY	0,41727194	1,4257208	0,043824703	0,24278243
HSA00980_METABOLISM_OF_XENOBIOTICS_BY_CYTOCHROME_P450	0,4347162	1,4202703	0,06921676	0,24626586
ST_INTERLEUKIN_4_PATHWAY	0,47865912	1,4153347	0,087037034	0,24933952

NAME	ES	NES	NOM p-val	FDR q-val
HSA00640_PROPANOATE_METABOLISM	0,6613602	2,168412	0	0,009259922
HSA00280_VALINE_LEUCINE_AND_ISOLEUCINE_DEGRADATION	0,62096816	2,1551082	0	0,006686125
VALINE_LEUCINE_AND_ISOLEUCINE_DEGRADATION	0,63490295	2,1063137	0	0,007488419
HSA00620_PYRUVATE_METABOLISM	0,60677165	2,1049461	0	0,005616314
HSA01032_GLYCAN_STRUCTURES_DEGRADATION	0,6298348	2,0116525	0	0,012953782
PROPANOATE_METABOLISM	0,6188834	2,0076818	0	0,010963367
HSA04514_CELL_ADHESION_MOLECULES	0,48748714	1,8817382	0,001677852	0,04114434
ERK5PATHWAY	0,65948033	1,8710899	0,003610108	0,040659428
HSA00980_METABOLISM_OF_XENOBIOTICS_BY_CYTOCHROME_P450	0,56329405	1,865157	0	0,03810608
BUTANOATE_METABOLISM	0,61255264	1,8642632	0,001923077	0,034708545
HSA04612_ANTIGEN_PROCESSING_AND_PRESENTATION	0,49442345	1,8133364	0,003683241	0,052842926
HSA02010_ABC_TRANSPORTERS_GENERAL	0,5851482	1,8062391	0,003552398	0,05115019
HSA04512_ECM_RECEPTOR_INTERACTION	0,4964385	1,7881804	0,005093379	0,054906245
HSA00650_BUTANOATE_METABOLISM	0,541188	1,7838193	0,010619469	0,05386255
HSA00071_FATTY_ACID_METABOLISM	0,5219613	1,7820176	0	0,050822698
GLUTAMATE_METABOLISM	0,59278804	1,7816434	0,007233273	0,047976706
HSA00903_LIMONENE_AND_PINENE_DEGRADATION	0,5560854	1,7593155	0,005395684	0,055301
CHOLESTEROL_BIOSYNTHESIS	0,63619506	1,7567546	0,005328597	0,05401572
HSA00330_ARGININE_AND_PROLINE_METABOLISM	0,550036	1,7355607	0,010544815	0,06148922
HSA00010_GLYCOLYSIS_AND_GLUCONEOGENESIS	0,47278315	1,6947418	0,012367492	0,0834043
BIOPEPTIDESPATHWAY	0,522466	1,6913894	0,010600707	0,08182326
HSA04670_LEUKOCYTE_TRANSENDOTHELIAL_MIGRATION	0,4327613	1,6888361	0,006578947	0,07937307
EDG1PATHWAY	0,5478723	1,6872157	0,00929368	0,07698762
HSA00251_GLUTAMATE_METABOLISM	0,5214958	1,6818556	0,013937282	0,0773043
PYRUVATE_METABOLISM	0,5046888	1,6764158	0,017636685	0,077361666
HSA04610_COMPLEMENT_AND_COAGULATION_CASCADES	0,5076563	1,6675353	0,011152417	0,080204695
HSA00512_O_GLYCAN_BIOSYNTHESIS	0,57678026	1,6672807	0,015414258	0,07734982
HSA00410_BETA_ALANINE_METABOLISM	0,5758374	1,6547607	0,025454545	0,08266963
GLUTATHIONE_METABOLISM	0,5240341	1,6373003	0,019366197	0,092778556
SIG_BCR_SIGNALING_PATHWAY	0,48485255	1,6324807	0,017452007	0,09292907
STARCH_AND_SUCROSE_METABOLISM	0,5431087	1,5987173	0,022847101	0,11416263
TRYPTOPHAN_METABOLISM	0,5384873	1,5767504	0,023593467	0,13106905
HSA00310_LYSINE_DEGRADATION	0,43180916	1,5682608	0,025252525	0,1355178
CITRATE_CYCLE_TCA_CYCLE	0,54384214	1,5543613	0,03130435	0,14506829
HSA00100_BIOSYNTHESIS_OF_STEROIDS	0,5021112	1,5541682	0,031141868	0,14110096
HDACPATHWAY	0,522617	1,5457045	0,023255814	0,14544147
HSA00530_AMINOSUGARS_METABOLISM	0,48527244	1,5407419	0,025684932	0,1462611
HSA00480_GLUTATHIONE_METABOLISM	0,47045353	1,5401647	0,03512015	0,14304417
PTENPATHWAY	0,5663444	1,5330625	0,030303031	0,14557333
SIG_PIP3_SIGNALING_IN_B_LYMPHOCYTES	0,480375	1,5315578	0,048780486	0,14342771
LYSINE_DEGRADATION	0,4825133	1,5275309	0,038938053	0,14353535
SPPAPATHWAY	0,53480667	1,4998771	0,04587156	0,1664887
HSA04650_NATURAL_KILLER_CELL_MEDIATED_CYTOTOXICITY	0,3727245	1,4825901	0,025996534	0,18185532
ST_INTERLEUKIN_4_PATHWAY	0,52482176	1,4791225	0,054770317	0,18200764
HSA00360_PHENYLALANINE_METABOLISM	0,5097929	1,4695456	0,072183095	0,18926553
HSA04920_ADIPOCYTOKINE_SIGNALING_PATHWAY	0,38913724	1,4651577	0,034013607	0,18981956
HSA00632_BENZOATE_DEGRADATION_VIA_COA_LIGATION	0,47845307	1,4646473	0,07594936	0,18640475
HSA04940_TYPE_I_DIABETES_MELLITUS	0,48073784	1,4604015	0,0680147	0,18707904
HSA00020_CITRATE_CYCLE	0,44804662	1,4586817	0,042735044	0,18530042
HSA00562_INOSITOL_PHOSPHATE_METABOLISM	0,4249757	1,4536787	0,050724637	0,18725881
HSA04664_FC_EPSILON_RI_SIGNALING_PATHWAY	0,3931928	1,4460684	0,041811846	0,19227923
ECMPATHWAY	0,48489302	1,4428461	0,06859206	0,19208598
HSA04510_FOCAL_ADHESION	0,32828078	1,44007	0,01821192	0,19132335
GHPATHWAY	0,5019717	1,4319124	0,076045625	0,19834845
CARBON_FIXATION	0,49022186	1,4181688	0,06238859	0,21060745
CREBPATHWAY	0,47175467	1,4096338	0,08514493	0,21831812
FMLPPATHWAY	0,4228648	1,4050558	0,06451613	0,22003631
HSA00252_ALANINE_AND_ASPARTATE_METABOLISM	0,44463888	1,403379	0,081597224	0,21818608
ST_B_CELL_ANTIGEN_RECEPTOR	0,43314153	1,4007052	0,07368421	0,21790797
ST_ADRENERGIC	0,44113365	1,3982333	0,09189189	0,21773982
HSA00710_CARBON_FIXATION	0,47635767	1,395614	0,07326008	0,21738192
GLYCINE_SERINE_AND_THREONINE_METABOLISM	0,45620888	1,3901086	0,09731544	0,22054277
HSA01430_CELL_COMMUNICATION	0,38053176	1,387499	0,061056107	0,21977004
ST_DICTYOSTELIUM_DISCOIDEUM_CAMP_CHEMOTAXIS_PATHWAY	0,4314962	1,385525	0,083484575	0,21890746
HSA00531_GLYCOSAMINOGLYCAN_DEGRADATION	0,49812272	1,3730905	0,10172745	0,23149928
PHOSPHATIDYLINOSITOL_SIGNALING_SYSTEM	0,35100484	1,3718354	0,060708262	0,2298458
INOSITOL_PHOSPHATE_METABOLISM	0,46246713	1,3636365	0,107344635	0,23688403
HSA04130_SNARE_INTERACTIONS_IN_VESICULAR_TRANSPORT	0,40691674	1,3616469	0,09174312	0,23640394
GLUCONEOGENESIS	0,383684	1,3558311	0,09413854	0,24126711
INTEGRIN_MEDIATED_CELL_ADHESION_KEGG	0,34797952	1,3538971	0,052892562	0,24068008

Gene	Primer	Sequence
HMGCR	F	AAACATTGTCACCGCCATCT
	R	GGGACCACTTGCTTCCATTA
HMGCoA Synthase1	F	CAAAAAGATCCATGCCCAGT
	R	TCAGCAACATCCGAGCTAGA
MVD	F	TGGCGGCAGTCACTTGTA
	R	GGCAGAACCAGCTCTTCATC
LSS	F	ATGCGCTCCTCAACCTGTAT
	R	GATGCTGATGCTCTTGGTGA
Actin	F	TGGGTATGGAATCCTGTG
	R	GGTCTTTACGGATGTCAAC
